# Flavonoids with Anti-Angiogenesis Function in Cancer

**DOI:** 10.3390/molecules29071570

**Published:** 2024-03-31

**Authors:** Qiang Wei, Yi-han Zhang

**Affiliations:** School of Medicine, Anhui Xinhua University, 555 Wangjiang West Road, Hefei 230088, China; zhangyihan@axhu.edu.cn

**Keywords:** flavonoids, plants, anti-angiogenesis, cancer, mechanism, signaling pathway

## Abstract

The formation of new blood vessels, known as angiogenesis, significantly impacts the development of multiple types of cancer. Consequently, researchers have focused on targeting this process to prevent and treat numerous disorders. However, most existing anti-angiogenic treatments rely on synthetic compounds and humanized monoclonal antibodies, often expensive or toxic, restricting patient access to these therapies. Hence, the pursuit of discovering new, affordable, less toxic, and efficient anti-angiogenic compounds is imperative. Numerous studies propose that natural plant-derived products exhibit these sought-after characteristics. The objective of this review is to delve into the anti-angiogenic properties exhibited by naturally derived flavonoids from plants, along with their underlying molecular mechanisms of action. Additionally, we summarize the structure, classification, and the relationship between flavonoids with their signaling pathways in plants as anti-angiogenic agents, including main HIF-1α/VEGF/VEGFR2/PI3K/AKT, Wnt/β-catenin, JNK1/STAT3, and MAPK/AP-1 pathways. Nonetheless, further research and innovative approaches are required to enhance their bioavailability for clinical application.

## 1. Introduction

The term “flavonoid” finds its roots in the Latin word “flavus”, which signifies the color yellow, as these compounds often manifest as secondary metabolites in a diverse range of plants [1]. Since the human body cannot synthesize flavonoids on its own, it is necessary to consume fruits, vegetables, or extracts from medicinal plants as a nutritional supplement. Many scientific researchers have showcased the pivotal role played by flavonoids in reducing the risk of cancer development viavarious mechanisms [2,3,4]. These mechanisms include safeguarding against DNA damage [5], inducing autophagy and apoptosis in tumor cells [6], inhibiting tumor cell invasion and metastasis [7], and suppressing angiogenesis [8], as well as modulating xenobiotic enzymes and antioxidant status [9].

The establishment of new blood vessels heavily accelerates the growth and metastasis of tumor cells, which provide essential blood support [10]. Consequently, angiogenesis inhibitors can impede tumor vascularization by inhibiting, regressing, or normalizing existing blood vessels. These inhibitors offer novel therapeutic approaches to hinder tumor growth, including the use of anti-angiogenic drugs comprised predominantly of antibodies (such as bevacizumab) and small molecule drugs (like sunitinib, sorafenib, and vandetanib) [11]. In general, tyrosine kinase (TK) receptors and their associated growth factors play a crucial role in stimulating endothelial cells to form new blood vessels. Thus, targeting these growth factors, their receptors, and related downstream signaling pathways has emerged as a promising strategy for drug discovery in angiogenesis inhibition [12]. For instance, anti-angiogenic antibodies selectively bind to VEGF and prevent its interaction with corresponding receptors [13]. On the other hand, small molecule agents act as tyrosine kinase inhibitors (TKIs) and directly target the VEGF receptor [14]. Additionally, cysteine proteases, like cathepsins L and B, have been linked to tumor growth and metastasis, playing roles in both intracellular proteolysis and extracellular matrix remodeling. Inhibition of cathepsins B or L also impedes the formation of new vascular structures [15].

In the quest for future cancer therapy drugs that are more potent, less toxic, and safer, the exploration of angiogenesis inhibitors becomes imperative. This review article delves into the crucial structural classifications and molecular mechanisms of 47 flavonoids from natural products, elucidating their antiangiogenic properties. It provides a comprehensive analysis of the distinct signal pathways through which these substances exert their antiangiogenic effects, offering a fresh perspective in this domain.

## 2. Flavonoids

**Figure 1:**
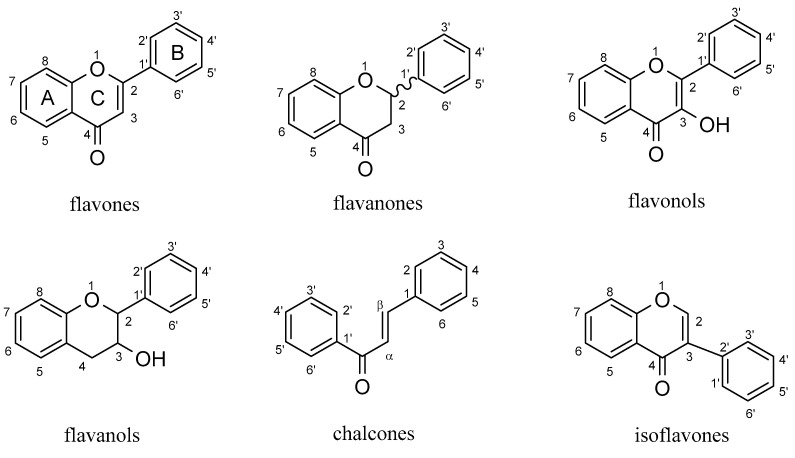
The partial structure of flavonoids.

Figure 1 illustrates flavonoids, a compound class distinguished by the attachment of two benzene rings to a central oxygenated ring C [16]. As depicted in Figure 1, research has identified six subclasses of flavonoids with antiangiogenic potential: flavones, flavanones, flavonols, flavanols, isoflavones, and chalcones. Flavones are characterized by a C-2 connected to ring B, highlighting a double bond of C-2 to C-3 [17]. Flavanones, also known as dihydroflavones, possess only a saturated, oxidized ring C. Much like flavones, flavonols possess a hydroxyl group linked to C-3 [18]. Flavanols share characteristics with flavones but have an unoxidized, saturated ring C with a hydroxyl group at C-3. Isoflavones are distinguished by a ring B attached to C-3 [16]. Finally, chalcones are the ring C-opening isomers of dihydroflavones and lack an oxygen-containing ring [16].

In order to explore the relationship between flavonoids and their anti-tumor vascular effects, we searched for the literature recorded in Google Scholar, Web of Science, and Pub Med to classify, summarize, and analyze the relation contents, including chemical structure of every flavonoid compound, its anti-angiogenesis mechanism in cancer, relational cancer models or cell lines, and the rise or fall of related indicators. Based on the study of pathological mechanism of cancer angiogenesis and flavonoids’ pharmacological action, we try to give flavonoids’ structure, classification, and signaling pathways as anti-angiogenic agents.

## 3. Results

### 3.1. Chemical Components

**Figure 2:**
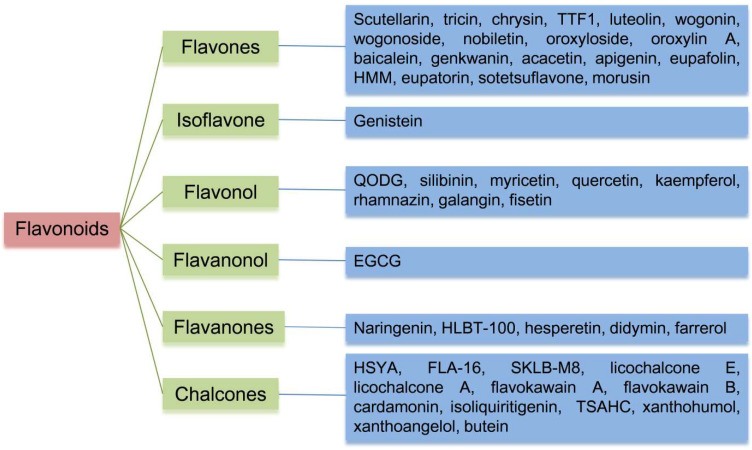
Flow diagram showing detailed classification of flavonoids.

**Figure 3:**
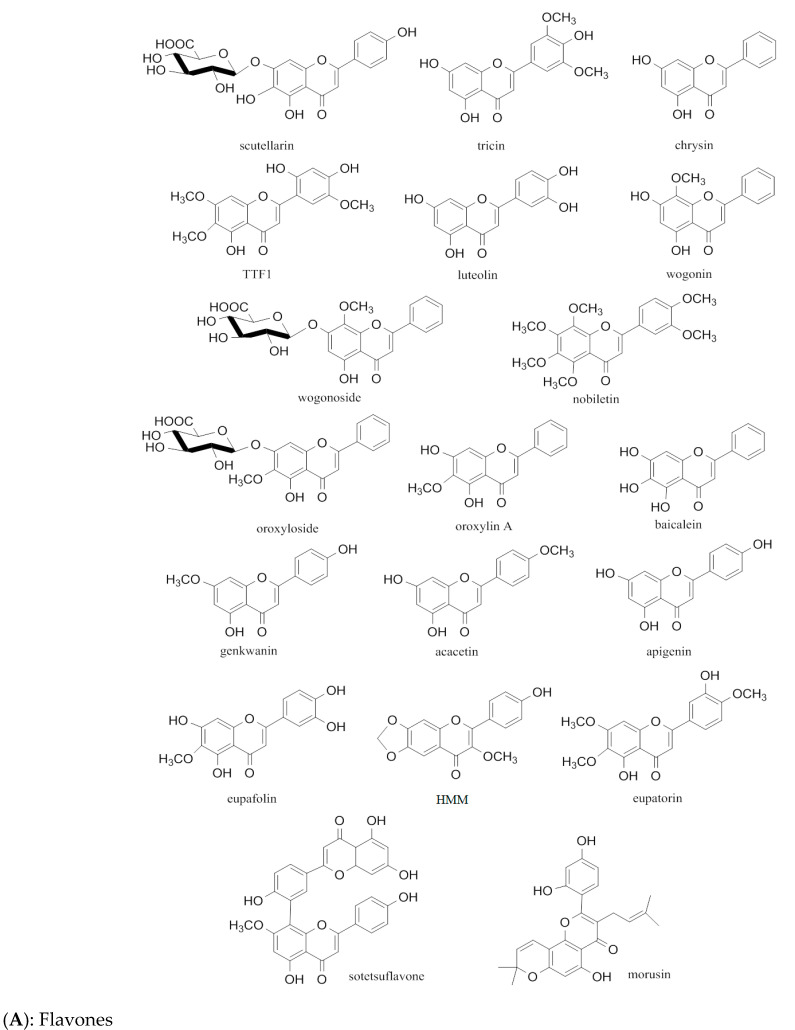

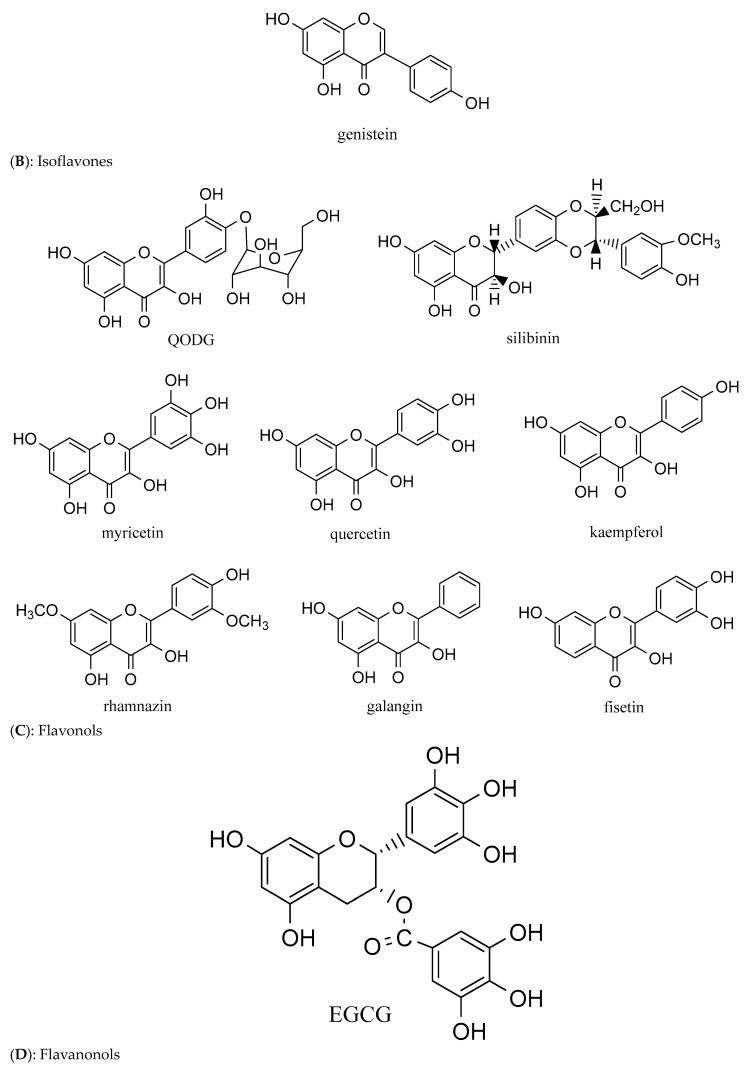

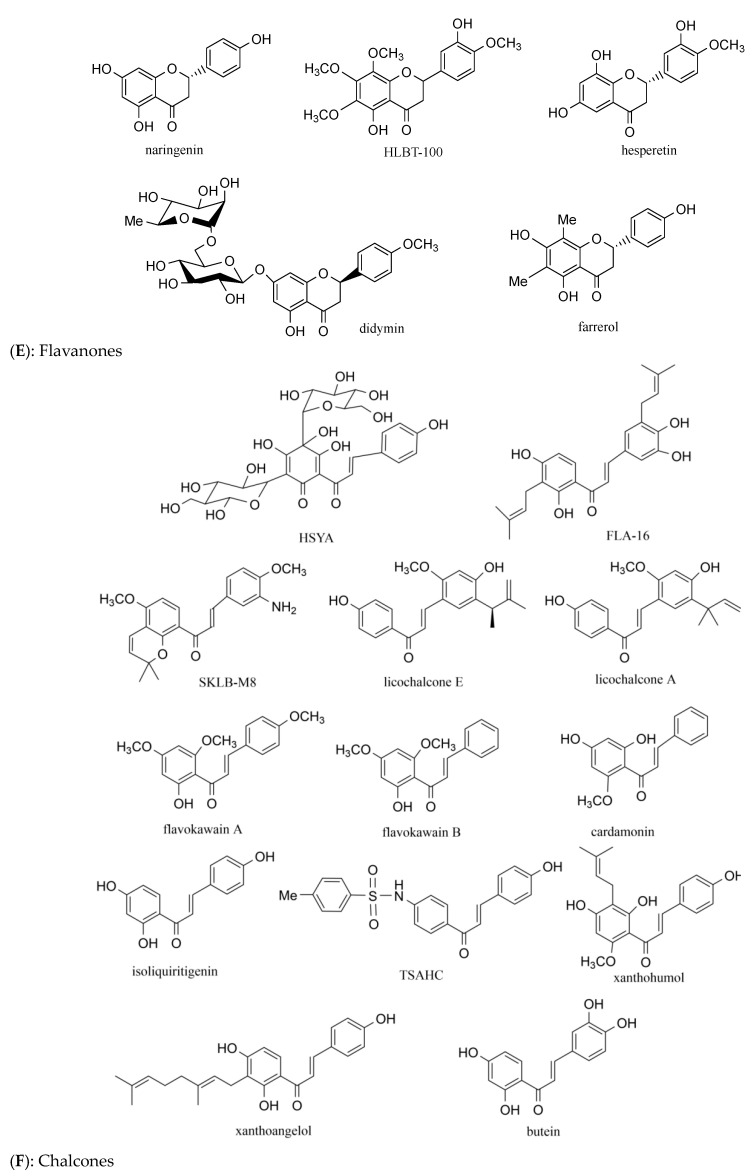
Structure of anti-angiongenic flavonoids in cancer.

There are a total of 47 flavonoids related to their antiangiogenic effects, and their classification and composition are shown in Figure 2 and Table 1, and their structure is shown in Figure 3. A more detailed table of statistics on antiangiogenic molecular mechanisms of different flavonoids can be found in Supplementary Materials.

### 3.2. Anti-Angiogenic Flavonoids in Cancer

#### 3.2.1. Flavones

Scutellarin (4′,5,6-trihydroxy-7-[(β-D-glucopyranuronosyl)oxy]flavone) is a compound found in Erigeron breviscapus. The VEGF signaling pathway regulates angiogenesis in endothelial cells viaephrinb2 and ephb4 [19]. In the context of colorectal cancer, scutellarin inhibits angiogenesis induced by cancer cells, migration of HUVECs, tube formation of HUVECs, and microvessel formation in chick embryo CAM. This inhibition is achieved by suppressing the expression of ephrinb2 [20]. Additionally, scutellarin may control the level of the transcription factor AP-1 to inhibit tumor angiogenesis in oral squamous cell carcinoma(OSCC), which is accomplished by decreasing the MMP-9 and MMP-2 levels, alongside decreasing integrin αvβ6 levels regarding human tongue cancer SAS cells. Notably, overexpression of integrin αvβ6 activates TGF-β, causing the generation of MMPs, cell migration, and survival [21]. Considering the crucial function of EMT activation in carcinoma metastasis, cell migration, invasion, and adhesion, scutellarin’s anti-angiogenic effects have been investigated in relation to its suppression of EMT, achieved by suppressing the PI3K/Akt/mTOR pathway [22], as seen in Table 1.

**Table 1:** Antiangiogenic molecular mechanisms of different flavonoids.

Type	Compounds (No.)	Mechanism	References
Flavone	Scutellarin (**1**)	Targeting ephrinb2 signaling; possibly regulating transcription factor AP-1; inhibiting PI3K/Akt/mTOR pathway	[20,21,22]
	Tricin (**2**)	Reducing ROS; inhibiting HIF-1αaccumulation	[23]
	Chrysin (**3**)	Regulating PI3K/Akt signaling; downregulating JAK1/STAT3 pathway and VEGF/VEGFR2 expression	[24,25,26]
	TTF1 (**4**)	Downregulating VEGF, KDR, bFGF, HIF-1α, and COX-2	[27]
	Luteonin (**5**)	Downregulating AEG-1, MMP-2, MMP-9, HIF-1α, and STAT3; stimulating immune response; inhibiting AKT/ERK/mTOR/P70S6K/MMPs pathway or PI3K/Akt/mTOR pathway; elevating JNK phosphorylation; inhibiting NF-κB-DNA binding activity; modulating IL-6/STAT3 pathway	[28,29,30,31,32,33,34,35,36]
	Wogonin (**6**)	Degrading HIF-1α protein; modulating c-Myc/HIF-1α/VEGF signaling axis; inhibiting VEGFR2phosphorylation or PI3K/Akt/NF-κB signaling	[37,38,39,40]
	Wogonoside (**7**)	Suppressing Wnt/β-catenin pathway	[41]
	Nobiletin (**8**)	Inhibiting VEGF- and bFGF-induced signaling; activating caspase pathway; inhibiting Akt phosphorylation; mediating Src/FAK/STAT3 signaling	[42,43,44]
	Oroxylin A (**9**)	Blocking KDR/Flk-1 phosphorylation	[45]
	Oroxyloside (**10**)	Inhibiting Akt/MAPK/NF-κB pathway	[46]
	Baicalein (**11**)	Partly mediating VEGF and FGFR-2 signalling; regulating p53/Rb signaling and TRAF6-mediated TLR4 pathway;inhibiting VEGF, HIF-1α, cMyc, NFκB, MMP-2, ROS, and PI 3K/Akt pathway, as well as ERK1/2 and p38 MAPK phospho-activation	[47,48,49,50,51,52]
	Genkwanin (**12**)	Inhibiting invasion and tube formation	[53]
	Acacetin (**13**)	Inhibiting AKT/HIF-1α pathway or STAT-VEGF axis	[54,55]
	Apigenin (**14**)	Blocking the ERK and ERK 1/2 survival signaling or IGF-I/IGFBP-3 signaling; regulating PI3K/AKT/p70S6K1 and HDM2/p53 pathways; downregulating HIF-1α, GLUT-1, and VEGF	[56,57,58,59]
	Eupafolin (**15**)	Blocking VEGFR2activation, ERK1/2, and Akt phosphorylation	[60]
	HMM (**16**)	Inhibiting cathepsins B and L	[61]
	Eupatorin (**17**)	Blocking the phospho-Akt pathway and cell cycle	[62]
	Sotetsuflavone (**18**)	Modulating PI3K/AKT and TNF-α/NF-κB pathways; inhibiting TGF-β, STAT3,and β-catenin; increasing endostatin and ZO-1	[63,64]
	Morusin (**19**)	Attenuating IL-6/STAT3 signaling; inhibiting VEGF and COX-2genes	[65,66]
Isoflavone	Genistein (**20**)	Suppressing autocrine and paracrine signalings, hypoxic activation of HIF-1, MMP-1, VEGF, PDGF-A, TF, uPA, MMP-2, and MMP-9; upregulating PAI-1, endostatin, angiostatin, TSP-1, CTGF, and CTAP; downregulating type IV collagenase, uPAR, protease M, PAR-2, VEGF, VEGFR, TGF-b, BPGF, LPA, TSP, JNK, and p38 activation; modulating TIMP-1 and -2 and PAI-1	[67,68,69,70,71,72]
Flavonol	Quercetin (**21**)	Regulating AKT/mTOR/P70S6K signaling; inhibiting NF-κB and MMP-2/MMP-9 signalings, the H-ras protein synthesis, VEGF and bFGF, STAT3 tyrosine phosphorylation, and Akt phosphorylation, NF-κB activity, eNOS, and early M-phase cell cyclearrest, p300 signaling and the binding of multiple transactivators to COX-2 promoter; upregulating TSP-1	[73,74,75,76,77,78,79,80]
	QODG (**22**)	Suppressing VEGFR2-mediated signaling	[81]
	Silibinin (**23**)	Downregulating survivin, VEGF, VEGFR-2, bFGF, NOS, COX, HIF-1α; increasing p53; inhibiting Akt and NF-kB signaling, MMP-2 secretion, PI3K/Akt signaling or Raf/MEK/ERK pathway, VEGF and endothelial cell growth, or NF-κB signaling; inducingapoptosis; upregulating VEGFR-1	[82,83,84,85,86,87,88,89,90]
	Myricetin (**24**)	Suppressing PI-3 kinase activity or PI3K/Akt/mTOR signaling; attenuating Akt/p70S6K phosphorylation; modulating Akt/p70S6K/HIF-1α/VEGF and p21/HIF-1α/VEGF pathways	[91,92,93]
	Kaempferol (**25**)	Regulating ERK-NF-κB-cMyc-p21-VEGF and VEGFR2 pathways, ERK/p38 MAPK and PI3K/Akt/mTOR pathways, Akt/HIF and ESRRA pathways; inhibiting VEGFR2 expression, VEGF and FGF pathways or PI3K/AKT, MEK, and ERK pathways	[94,95,96,97,98]
	Rhamnazin (**26**)	Regulating VEGF and PEDF; downregulating the VEGFR2/STAT3/MAPK/Akt pathway	[99,100]
	Galangin (**27**)	Downregulating CD44 and VEGF; modulating Akt/p70S6K/HIF-1α/VEGF pathway	[92,101]
	Fisetin (**28**)	Inhibiting MMPs, MMP-8, and MMP-13, p38 MAPK-dependent NF-κB pathway, NF-κB, MAPK, Wnt, Akt, and mTOR; G1 phrase-G2/M arrest; downregulating cyclin D1, survivin, VEGF, eNOS, iNOS, Bcl-2; inducing p53 and p21, Bax expression and cleavage of caspases-3 and -7, and PARP; regulated by HO-1 viatranscription factor Nrf2; inactivating PI3K/Akt and JNK pathways; diminishing NF-κB and AP-1 DNA-binding activities	[102,103,104,105,106,107,108,109,110,111,112]
Flavanonol	EGCG (**29**)	Inhibiting VEGF-induced VEGFR2 signaling or NF-κB and ERK1/2 signalings, endoglin/pSmad1 signaling, DNA synthesis, cell proliferation, and signal transduction pathway, and PI3K/AKT/mTOR/HIF1α pathway; downregulating VEGF, uPA, angiopoietin 1 and 2, VEGFR-1 and -2, ERK-1 and -2, MMP-2 and -9, HIF-1α, and CXCL12; suppressing HIF-1 and VEGF/VEGFR axis activation, VEGF, IL-8, and CD31 and Akt activation, NF-κB, and MT1-MMP; increasing endostatin and TIMP1; modulating the genes transcription	[113,114,115,116,117,118,119,120,121,122,123,124,125,126,127,128,129,130,131,132,133]
Flavanones	Naringenin (**30**)	Mediating ERRα/VEGF/KDR signaling	[134]
	HLBT-001 (**31**)	Not mentioned	[135]
	Hesperetin (**32**)	Modulating PI3K/AKT, ERK and p38 MAPK signalings	[136]
		Inhibiting angiogenic growth factors and COX-2 mRNA expression	[137]
	Didymin (**33**)	Preventing NF-κB and expression of adhesion molecules	[138]
	Farrerol (**34**)	Downregulating Akt/mTOR, Erk and Jak2/Stat3 signalings	[139]
Chalcone	HSYA (**35**)	Inhibiting tumor vascularization; blocking ERK/MAPK and NF-κB signaling or p38 MAPK phosphorylation; downregulating VEGF, bFGF and MMP-9	[140,141,142,143,144]
	FLA-16 (**36**)	Modulating PI3K/Akt signaling through the inhibition of CYP4A	[145]
	SKLB-M8 (**37**)	Decreasing ERK1/2 phosphorylation	[146]
	LicA (**38**)	Blocking VEGF/VEGFR-2 signaling	[147]
	LicE (**39**)	Decreasing VEGFR2, VEGF-A, HIF-1α, COX-2 and iNOS	[148]
	FKB (**40**)	Reducing angiogenin, F3, SDF-1, serpin F1, and TSP-2; suppressing the formation of vessels	[149,150]
	FKA (**41**)	Inhibiting new blood vessels; downregulating the androgen receptor	[151,152]
	Cardamonin (**42**)	Inhibiting HIF-α and VEGF; regulating ERK1/2 and AKT signaling; downregulating miR-21	[153,154,155]
	Isoliquiritigenin (**43**)	Hampering MAPK signaling of JNK and p38, VEGF/VEGFR2 pathway, ERK1/2 and VEGF; promoting PEDF expression or JNK	[156,157,158,159,160]
	TSAHC (**44**)	Disturbing protein–protein interaction between TM4SF5 and other membrane receptors	[161]
	Xanthohumol (**45**)	Mitigating NF-κB activity, AMPK and AKT/mTOR pathways, and Akt/NF-kB signaling; modulating NF-κB signalling; inhibiting ICAM-1, MMP-9, VEGF, and NF-κB activity	[113,114,115,116,162,163,164]
	Xanthoangelol (**46**)	Inhibiting tube formationand the binding of VEGF to vascular endothelial cells	[117]
	Butein (**47**)	Targeting the AKT/mTOR translation-dependent signaling; inhibiting NF-κB signaling	[118,119]

Tricin, namely 4′,5,7-trihydroxy-3′,5′-dimethoxyflavone, is a substance found in some foods such as rice and wheat [120]. Inhibitory effects of tricin on the proliferation and invasion triggered by VEGF, as well as HUVEC tube assembly and angiogenesis of CAM. These effects are achieved by downregulating the signal transduction of VEGFR2, partially through diminishing the generation of ROS in endothelial cells. Additionally, tricin inhibits the VEGF expression by preventing the accumulation of HIF-1α in tumor cells [23].

Chrysin (5,7-dihydroxyflavone) is a bioactive component available in diverse fruits, vegetables, and mushrooms [165]. Dampened activities of chrysin on angiogenesis and the HIF-1α level in DU145 prostatic carcinoma cells is achieved viathe PI3K/Akt pathway. Boosting the oxygen-controlled degradation domain prolyl hydroxylation, chrysin triggers increased ubiquitin tagging and degradation of HIF-1a, simultaneously interfering with a HIF-1α interacting a heat shock protein 90 (HSP90) [24]. Another study reveals that the anti-angiogenic effects of chrysin are associated with the inhibition of sIL-6R, gp130, phosphorylated JAK1, STAT3, and VEGF expression in HUVECs and CAM assay [25]. Flavonoids are known to inhibit inflammation-induced angiogenesis, and chrysin specifically inhibits lipopolysaccharide (LPS)-induced CAM neovascular density, downregulating VEGF/VEGFR2 expression, and disrupting an IL-6/IL-6R self-regulatory loop using HUVECs [26].

Isolated from Sorbariasorbifolia, TTF1, also known as 5,2′,4′-Trihydroxy-6,7,5′-trimethoxyflavone, has showcased its capacity to inhibit tumor angiogenesis elicited by HepG-2 cells via reducing the VEGF, KDR, bFGF, COX-2, and HIF-1α levels of RNA and protein in key factors regulating angiogenesis [27].

Luteolin (3′,4′,5,7-tetrahydroxyflavone), a compound commonly existing in medicative plants or veggies [121], been demonstrated to exhibit potent anti-angiogenic effects on the chick chorioallantoic membrane and to possess anti-invasive activity against breast cancer cells. It achieves this by downregulating the expression of various angiogenesis-related factors, including dampening the MMP-2 and astrocyte elevated gene 1 (AEG-1) expression [28]. Furthermore, it suppresses angiogenesis through the decrease expression of VEGF-A and MMP-9 [29]. Similar effects on tumor angiogenesis have been observed with luteolin, as it reduces phosphorylated VEGFR2 induced by VEGF-A and inhibits subsequent proteins including mTOR, AKT, P70S6K, ERK, MMP-2, and MMP-9 [30]. In addition, luteolin exerts its action of anti-angiogenesis by diminishing VEGF secretion through mitigating VEGF mRNA production, which is regulated by inhibiting the NF-κB transcription activity [31]. Gas6 initiates the activation of Axl receptor tyrosine kinase (Axl), which, in turn, drives the Gas6/Axl pathway. This pathway not only fosters growth, movement, infiltration, and tubulogenesis but also further governs the PI3K/Akt/mTOR pathway [32]. Luteolin also inhibits the Gas6/Axl pathway and its downstream PI3K/Akt/mTOR pathway in HMECs-1 in vitro, thus exerting an anti-angiogenic effect [33]. The activated STAT3 protein contributes to cell growth, differentiation, and the upregulation of VEGF expression, leading to induction of tumor angiogenesis [34]. Luteolin exhibits anti-angiogenic effects by inhibiting the HIF-1α and phosphorylated STAT3 (p-STAT3) pathways, particularly within the alternatively activated TAMs [35]. Additionally, luteolin exerts inhibitory effects viathe IL-6/STAT3 pathway, which modulates the level of IL-6 receptor (IL-6Rα), resulting in decreased MMP-2 levels and increased the protein expression of suppressor of cytokine signaling (SOCS3) [36]. SOCS3, regulated by STAT3, negatively regulates JAK activation and IL-6-mediated signaling, thereby impacting the JAK/STAT signaling [122].

Obtained from Scutellariabaicalensis, wogonin (5,7-dihydroxy-8-methoxyflavone) has demonstrated an ability to suppress angiogenesis. It achieves this by decreasing the HIF-1α level and increasing PHD1, 2, 3 expression, as well as VHL E3 ubiquitin ligase. This, in turn, reduces the heatshock protein 90 (Hsp90), client proteins such as EGFR, Cdk4, and survivin, leading to their degradation in the proteasome. Additionally, wogonin hinders the binding between Hsp90 and HIF-1α, resulting in the downstream reduction inVEGF secretion [37]. Angiogenesis is closely linked to the progression of multiple myeloma (MM). Wogonin is shown to suppress MM-stimulated angiogenesis and reduce levels of secreted VEGF, platelet-derived growth factor (PDGF), and bFGF by way of the c-Myc/HIF-1α/VEGF pathway [38]. Further examination of the signaling pathway reveals that wogonin hinders HUVEC migration and tube assembly triggered by VEGF and blocks VEGF-induced tyrosine phosphorylation of VEGFR2. Moreover, significant decreases inAKT, ERK, and p38 phosphorylation induced by VEGF are observed [39]. In addition, wogonin manifests inhibitory action on H_2_O_2_-induced angiogenesis in HUVECs based on the suppression of the signaling pathway of PI3K/Akt and NF-κB [40]. Another flavone glycoside derived from Scutellariabaicalensis Georgi, called wogonoside (wogonin-7-*O*-glucuronide), has also exhibited anti-angiogenic activity by suppressing the Wnt/β-catenin signaling in MCF-7 cells. This results in reduced intracellular levels of Wnt3a, an increase in GSK-3β and AXIN expression, and enhanced β-catenin phosphorylation, facilitating its degradation by the proteasome [41].

Nobiletin (5,6,7,8,3′,4′-hexamethoxyflavone), a polymethoxyflavonoid present in specific citrus fruits [123], has demonstrated the ability to hinder various endothelial cell functions and angiogenesis by thwarting the phosphorylated ERK1/2 and JNK induced by FGF in HUVECs [42]. Moreover, nobiletin has shown the capability to hinder cancerous growth and angiogenic processes by reducing AKT activation, consequently inhibiting VEGF, NF-κB, and HIF-1α. By downregulating AKT, HIF-1α secretion is inhibited, leading to the suppression of VEGF in ovarian cancer cells [43]. Additionally, nobiletin has been discovered to impede cancer angiogenesis in mammary carcinoma showing estrogen receptor positivity by restraining the signaling mediated by Src, FAK, and STAT3 while fostering PXN gene expression [44].

Oroxylin A (5,7-dihydroxy-6-methoxyflavone) is a prominent bioactive flavonone found in the roots of Scutellariabaicalensis. It effectively exerts antiangiogenic activity by significantly inhibiting the phosphorylated VEGFR2 induced by VEGF as well as its subsequent signaling factors such as Akt, p38 MAPK, and ERK1/2 [45]. Oroxyloside, known as Oroxylin A 7-*O*-glucuronide, one of the primary metabolites of oroxylin A, also exhibits anti-angiogenic properties. It suppresses the autophosphorylation of VEGFR2/Flk-1 and upregulates the expression of R-Ras and E-cadherin by inhibiting the downstream Akt/MAPK/NF-κB pathways, leading to a decrease inthe process that proteins translocate into the nucleus and NF-κB’s capacity to bind DNA [46].

Derived from Scutellariabaicalensis Georgi, baicalein, also called 5,6,7-trihydroxyflavone [124]. Its anti-angiogenesis activity is attributed to the VEGF suppression and FGFR-2, and the downregulation of corresponding factors such as FGF2, VEGF, MMP1, TEK, and ANGPT1 [47]. Baicalein also effectively suppresses MMP-2 activity associated with cells, thereby inhibiting migration, proliferation, and in vitro capillary formation in bFGF-treated HUVECs [48]. Furthermore, baicalein has exhibited the ability to hinder angiogenesis via multiple pathways, reducing expression levels of HIF-α, VEGF, NF-κB, and c-Myc in ovarian cancer cells [49], decreases the hypoxia-induced genes expression of hypoxia-responsive COX-2, VEGF, and iNOS by hindering the PI3K/Akt and ROS signaling in BV2 microglia [50], and attenuates the phosphorylation of VEGF2 and ERK in baicalein-treated HUVECs by lowering G1-related proteins expression, leading to stalling the cell cycle at the G1/S boundary, and affecting the p53/Rb signaling [51]. Additionally, this leads to a notable reduction in TRAF6 triggered by LPS and the phosphorylation of ERK, AKT, and p38, further inhibiting HUVECs proliferation, migration, and tube-like structure generation [52]. Overall, baicalein demonstrates diverse anti-angiogenesis properties and regulates multiple molecular pathways to impede angiogenesis in various contexts.

Genkwanin (4′,5-dihydroxy-7-methoxyflavone) is a compound found in various plants, including Alnus Glutinosa (Betulaceae) [125], Leonurus turkestanicus [126], and Thymus taxa [127]. Studies have shown that genkwanin exhibits a stronger inhibitory effect on angiogenesis stimulated by VEGF in HUVECs compared to apigenin, showing efficiently the inhibition of invasion and tube formation without affecting endothelial cell viability [53].

Acacetin (5,7-dihydroxy-4′-methoxyflavone), isolated from various plants, seeds, and flowers, has demonstrated the ability to suppress VEGF level via activating AKT and degrading subsequent its downstream target, such as HIF-1α protein [54]. This inhibitory effect is also mediated by blocking the STAT/VEGF pathway in both tumor cells and endothelial cells, leading to a decrease in phosphorylated STAT-1 and phosphorylated STAT-3, along with a downregulation of angiogenesis-promoting factors like VEGF, bFGF, iNOS, eNOS, and MMP-2 in HUVECs [55].

Apigenin (5,7,4′-trihydroxyflavone) is a widely encountered flavone from various fruits and veggies such as parsley, grapes, and apples [128]. It has demonstrated significant anti-angiogenic effects, potentially achieved by inhibiting vascular tube assembly and inducing apoptotic process in endothelial cells assembling tubes. This mechanism entails the suppression of ERK 1/2-mediated cell survival pathway and accompanied by the downregulation of cell adhesion molecules associated with angiogenesis, involving vascular PECAM-1and E-cadherin. Apigenin additionally triggers caspase-3 activation, leading to the cleavage of PARP and lamin A/C [56]. In addition, apigenin exerts its anti-angiogenic activity by suppressing VEGF and HIF-1α levels viathe PI3K/AKT/p70S6K1 and human murine double minute 2 (HDM2)/p53 pathways [57]. It downregulates the levels of HIF-1α, GLUT-1, and VEGF in pancreatic carcinoma [58] and inhibits the level of MMP-2, MMP-9, uPA, and VEGF in TRAMP mouse by downregulating IGF-I/IGFBP-3 signaling [59].

Eupafolin (5,7,3′,4′-tetrahydroxy-6-methoxy-flavone) is a flavone compound that can be found in various plants including Artemisia princeps Pampanini [129], Eupatorium litoralle [130], and Phyla nodiflora [131]. Studies have shown that eupafolin hampers tumor cell growth, movement, and tubular structure development induced by VEGF bycurbing the VEGFR2 activation, leading to reduced phosphorylation of ERK1/2 and Akt signaling pathways [60].

Cathepsin B is an enzyme that is predominantly found in microvascular endothelial cells surrounding human glioblastoma and prostate carcinomas [132]. It significantly influences the MMPs and TIMPs balance. By inactivating TIMP-2 and TIMP-3, cathepsin B shifts this balance, thereby promoting an angiogenic environment [133]. Another cathepsin enzyme, cathepsin L, is upregulated in various human cancers. It interacts with the glycosaminoglycan components of proteoglycans present on cell surfaces and extracellular matrices, leading to enhanced tumor metastasis and angiogenesis [166]. A novel flavone called 4′-Hydroxy-6,7-methylenedioxy-3-methoxyflavone(HMM), isolated from Dulaciaegleri, has demonstrated anti-angiogenic effects by specifically inhibiting the cysteine proteases cathepsins B and L [61].

Eupatorin (3′-hydroxy-5,6,7,4′-tetramethoxyflavone), which is obtained from Orthosiphon stamineus [167], has been found to inhibit the emergence of neovascularization and the process of angiogenesis in mouse aorta. This effect is achieved by blocking the phospho-Akt pathway in human breast cancer cells. Additionally, eupatorin demonstrates a correlation with the downregulation of Bcl2L11, VEGFA, and HIF1A genes, further supporting its anti-angiogenic properties [62].

Sotetsuflavone (7-*O*-methylamentoflavone), a biflavone obtained from Selaginella denticulata [168] or Torreyayunnanensis [169], has demonstrated the ability to dwindle VEGF level in A549 cells due to an increase in angiostatin expression by reducing HIF-1α levels and downregulating TNF-α, thereby inhibiting NF-κB expression [63]. Moreover, sotetsuflavone exhibits an effective anti-angiogenic result using the inhibition of TGF-β, STAT3, and β-catenin expression, while simultaneously upregulating the levels of endostatin and ZO-1 [64].

Morusin, an isoprenylated flavone derived from Morus alba Linn. [170], demonstrates significant constricting effects on migration, invasion, and angiogenesis in hepatocellular carcinoma cells (HepG2 and Hep3B) as well as HUVECs. This inhibition correlates with a decrease inkey factors, including VEGFR2, VEGF, MMP9, and MMP2, by effectively suppressing the IL-6-mediated STAT3 pathway [65]. Furthermore, it effectively reduces the expression of VEGF and COX-2 genes, both of which are implicated in angiogenesis of A549 cells [66].

#### 3.2.2. Isoflavone

Genistein (4′,5,7-trihydroxyisoflavone), a soy phytoestrogen, has been the subject of multiple studies revealing its significant ability to suppress the baseline and hypoxia-triggered VEGF level in HUVECs and prostate cancer. This suppression is associatedwithareduction inHIF-1α nuclear gathering [67]. In pancreatic carcinoma cells, genistein inhibits the activated procedure of HIF-1 induced by hypoxia, resulting in the inhibition of hypoxia-mediated upregulation of VEGF gene expression [68]. Furthermore, genistein demonstrates anti-angiogenic properties by partly blocking the uPA and MMP-1 expression together with the activated pro-MMP-2 stimulated by VEGF/bFGF, via modulating the secretion of their inhibitors of PAI-1, TIMP-1, and TIMP-2 [69]. In human bladder cancer cells, genistein suppresses production or release of angiogenic proteins of PDGF-A, tissue factor (TF), and VEGF165, as well as enzymes involved in matrix degradation for MMP-2, MMP-9, and uPA. Additionally, it boosts the level of PAI-1, angiostatin, TSP-1, and endostatin as anti-angiogenic agents [70]. The anti-angiogenic activity of genistein is also investigated in prostate cancer, where it downregulates the genes level of uPAR, MMP-9, VEGF, neuropilin, TSP-1, TSP, TGF-β2, BPGF, PAR-2, protease M, and LPA. Conversely, it upregulates the expression of genes such as CTAP and CTGF [71]. Moreover, genistein inhibits the production and effect of p38, MMP-9, JNK, and MMP-2 viathe inhibition of the activities of PTK and MAPK in VEGF-stimulated endothelial cells [72].

#### 3.2.3. Flavonol

Quercetin (3,3′,4′,5,7-pentahydroxyflavone), a flavonol derived from various vegetables and fruits [171], has exhibited anti-angiogenesis properties viamodulation of the AKT/mTOR/P70S6K signaling cascade regulated by VEGFR2 [73]. It also hinders MMP-2 and MMP-9 signaling through curbing the MAPK and PI3K/AKT signaling cascades [74]. Moreover, it inhibits VEGF and bFGF expression, leading to MVD reduction, and suppresses the synthesis of H-ras protein, consequently halting cancerous cell proliferation and angiogenesis in a mammary carcinoma model induced by DMBA [75]. Quercetin also upregulates the TSP-1 level as a factor hindering angiogenesis [76], inhibits hypoxia-induced VEGF expression by suppressing STAT3 tyrosine phosphorylation viaan alternative mechanism independent of nuclear HIF levels, inducing HIF-1a expression under hypoxic conditions [77], substantially suppresses VEGF expression by inhibiting NF-κB activity [78], and hampers eNOS phosphorylation and early M-phase cell cycle arrest [79]. Additionally, the inhibition of COX-2-mediated angiogenesis by quercetin links to impeding COX-2 generation mainly regulated by hindering p300 HAT function [80]. Alternatively, quercetin-4′-*O*-β-D-glucopyranoside (QODG) found in Hypericum attenuatum, has been proved to inhibit angiogenesis in HUVECs by suppressing the phosphorylated VEGFR2 triggered by VEGF, leading to the inhibition of subsequent kinases of p70S6K, c-Src, AKT, FAK, mTOR, and ERK [81].

Silybum marianum contains a flavonol called silibinin, also known as silibinin, namely,5,7-trihydroxy-2-[3-(S)-(4-hydroxy-3-methoxyphenyl)-2-(S)-(hydroxy-methyl)-2,3- dihydro-1,4-benzodioxin-6-yl]chroman-4-one) [172]. It has been found to inhibit tumor angiogenesis in RT4 xenografts by downregulating survivin and increasing p53 expression [82]. Silibinin’s anti-angiogenic effects are connected with a decline in VEGF secretion and an enhancement in VEGFR1 gene expression [83]. Similarly, the downregulation of VEGF and improvement of IGFBP-3 lead to an inhibitory effect on the mitogenic action of IGF-I [84]. Silibinin also demonstrates a defense activity against sunlight-induced skin cancer by downregulating angiogenic response, involving gene-regulating proteins of NF-κB, COX-2, STAT3, HIF-1α, iNOS, and their potential upstream regulators, p-STAT3 and phospho-p65 in skin cancer triggered by ultraviolet [85]. Additionally, it decreases the levels of VEGF and VEGFR2 expression while attenuating circulating levels of bFGF [86]. Silibinin also reduces the expression of VEGF, COX-2, COX-1, NOS, HIF-1α, and NOS3 [87]. It further inhibits the secretion of MMP-2, possibly entailing the downregulation of survivin and hindrance of the NF-κB together with Akt pathways, in which NF-kB activation is not dependent on the Akt pathway and has been found to be associated with LY294002 targets other than PI3K in HUVECs [88]. Moreover, silibinin blocks the accumulation of HIF-1α and hypoxia-induced VEGF secretion by downregulating the mTOR/p70S6K/4E-BP1 signaling [89]. Another study indicated that silibininprevents the levels of VEGF and MMP-9 stimulated by TPA viathe restraint of the Raf/MEK/ERK signaling [90].

Myricetin (3,5,7,3′,4′,5′-hexahydroxyflavone) exists in various berries, vegetables, walnuts, tea, onions, grapes, and medicinal herbs [173,174,175]. It has been shown to inhibit ultraviolet B-induced angiogenesis by blocking the expression of HIF-1α viadirect inhibition of PI3K activity, which is a critical target. This inhibition leads to a decrease in the level of MMP-9, VEGF, and MMP-13 [91]. The anti-angiogenic effects of myricetin are attributed, at least in part, to its effects on the VEGF, HIF-1α, p70S6K, and Akt signaling, resulting in a reduction in VEGF secretion and proteins expression of HIF-1α, p-Akt, and p-70S6K. Additionally, it has been discovered that myricetin hinders angiogenesis via an unconventional signaling that encompasses HIF-1α, VEGF, and p21 in OVCAR-3 cells [92]. Myricetin also exerts antiangiogenic effects by inhibiting cell migration and tube formation, triggering apoptosis induced by ROS while suppressing the signaling factors of Akt, PI3K, mTOR [93].

Kaempferol (3,5,7,4′-tetrahydroxyflavone), which is abundant in vegetative foods [176], shows the inhibitory effect on VEGF and angiogenesis by regulating the signaling factors of VEGF, cMyc, NF-κB, ERK, and p21. It downregulates the phosphorylated ERK, and the levels of cMyc and NF-κB while promoting p21 expression [94]. Another study also reported that kaempferol’s anti-angiogenic effect and inhibition of VEGF level is performed by reducing HIF-1α expression via two different pathways, Akt/HIF and ESRRA, in which ESRRA, an orphan nuclear receptor, shares significant sequence similarity and engages in intense crosstalk with estrogen receptors [95]. Additionally, kaempferol was found to significantly suppress both VEGF and FGF signaling pathways, leading to a direct reduction in VEGFR2 expression [96]. Moreover, kaempferol lowers VEGF/VEGFR2 levels and diminishes its subsequent signaling, such as AKT, MEK1/2, p-mTOR, ERK1/2,and PI3K [97]. It additionally blocks the activated process of mTOR, Akt, and the subsequent p70S6K effector. Furthermore, kaempferol decreases the activated VEGFR2 and HIF-1α in endothelium and phosphorylated p38, Akt, mTOR, and ERK, thereby reducing VEGFR2 and HIF-1αphosphorylation viathe restriction of ERK/p38 MAPK and PI3K/Akt/mTOR signaling pathways [98].

Rhamnazin (7,3′-dimethoxy-3,5,4′-trihydroxyflavone) in therapeutic botanicals such as Ginkgo biloba [99], has shown potent anti-angiogenic activities in neovascularization after alkaline burn. It directly reduces the VEGF-stimulated phosphorylation of VEGFR2, leading to the restraint of downstream signaling of MAPK, STAT3, and AKTwithout affecting their total levels [99,100].

Extensive evidence has compellingly demonstrated that CD44, known for its vigorous angiogenic properties, is critical for angiogenesis through its modification of VEGF. This makes CD44 an important therapeutic target in the context of glioblastoma [101]. Galangin (3,5,7-trihydroxyflavone), which is found in galagal root, india root, and Alpinis officinarum [177], exhibits inhibitory effects on angiogenesis by downregulating VEGF expression in HUVECs [101]. Similar to myricetin’s anti-angiogenic properties, galangin suppresses VEGF expression and downregulates p-Akt, p-p70S6K, and HIF-1α levels, contributing to its inhibitory effect on angiogenesis [92].

Fisetin (3,7,3′,4′-tetrahydroxyflavone) in strawberries, grapes, apples, onions, and cucumbers [178]. It exhibits anti-angiogenic activity by suppressing the activity of MMPs [102]. Specifically, it inhibits MMP-1, MMP-3, MMP-7, MMP-9, and MMP-14, which hinders the expansion of HUVECs and the activated process of proMMP-2 regulated by MMP-14 in human fibrosarcoma cells [102]. Based on a molecular docking method, it also inhibits post-translational forms of MMP-8 and MMP-13 in colorectal cancer progression and significantly palliates colorectal cancer invasion and metastasis [103]. Furthermore, fisetin suppresses HUVEC cell migration and VEGF-induced conditions by inducing G1 phase arrest and mild G2/M arrest. It also inhibits the level of cyclin D1, survivin, VEGF, Bcl-2, eNOS, iNOS, and MMPs and elevates the level of caspases-3, caspases-7, PARP, p53, p21, and Bax in prostate carcinoma and lung cancer [104,105]. The HO-1 elevation has been associated with tumor angiogenesis [179]. Fisetin inhibits cell migration in mammary carcinoma by silencing the gene regulatory protein Nrf2 in the nuclear fraction, leading to reduced activity of MMP-9 and MMP-2 [106]. Similar downregulation of MMP-2 and MMP-9 levels has been observed in prostatic carcinoma by inhibiting the signaling of JNK, PI3K, Akt to reduce NF-κB and AP-1 DNA-binding activities [107]. uPA is involved in matrix degradation, migration, invasion, metastasis, and tumor angiogenesis [180,181]. Fisetin partially suppresses the uPA-dependent increase in cell migration and invasion by inhibiting p38 MAPK activation. This inhibition is achieved byreducing the translocation of p38 MAPK to the nucleus and decreasing NF-κB’s DNA-binding activities in adenocarcinoma of the cervix. Consequently, uPA expression is downregulated [108]. Additionally, Death receptor 3 (DR3), a TNF family member acting as a receptor for TNF, is a cell surface protein that has been revealed to induce NF-κB activation when overexpressed in animal cells. NF-κB is known to provoke angiogenesis and metastasis viathe regulation of VEGF and MMPs. Inhibition of NF-κB signaling in pancreatic carcinoma is attributed to the inhibition of DR3-mediated NF-κB activation [109,110]. Fisetin’s inhibition of uPA enzyme in the capillary vessels surrounding the tumor could be involved in areduction inangiogenesis and subsequently impede neoplastic expansion [111,112].

#### 3.2.4. Flavanonol

Epigallocatechin-3-gallate (EGCG), from tea, demonstrates anti-angiogenic activity by inhibiting VEGF-induced VEGFR2 signaling in HUVECs. This effect is attributed to the direct interaction between EGCG and the VEGF peptide [182,183]. EGCG also suppresses VEGF expression, hinders the attachment between growth factor with VEGFR2, or impedes the receptor’s phosphorylation process, showing the suppressing the internal cellular signaling and stimulation of mitosis triggered by VEGF [184,185,186]. These additional actions result in increased endostatin expression and inhibition of VEGF mediated by EGCG [187]. Furthermore, stimulating the VEGF/VEGFR pathway leads to lowered protein levels such as HIF-1α protein, heregulin mRNAs, etc., as well as subsequent expressions of VEGFR2, p-VEGFR2, in SW837 colorectal malignancy cells [188]. EGCG exhibits comparable effects on angiogenesis in lung cancer cells. In both cases, it suppressed the secretion of HIF-1α protein, and CD31, VEGF, and IL-8 were triggered by HIF-1α while also activating Akt [189].

EGCG also exerts inhibitory effects on VEGF level and secretion triggered by IL-6, as well as gastric carcinoma cells’ angiogenesis by suppressing STAT3 activity. This ultimately targets the STAT3/VEGF signaling pathway, which leads to the declined VEGF level induced by IL-6. This is attained byinhibiting the process of STAT3 translocation, access to nucleus and combination with VEGF promoter in gastric carcinoma [190,191], as well as the inhibition of VEGF protein level, secretion, and mRNA expression due to reduced activation of STAT3 in gastric carcinoma [192].

In A549 cells, nicotine enhances related proteins levels, such as VEGF, COX-2, HIF-1α, p-Akt, etc. However, the compound EGCG downregulates these expressions, leading to the suppression of HIF-1α-induced angiogenesis [193]. In human prostate carcinoma LNCaP cells, EGCG induces anti-angiogenesis by suppressing the expression of VEGF, angiopoietin 1 and 2, etc., and increasing the level of TIMP1, in which angiopoietins (specifically, angiopoietin 1 and 2) are signaling proteins that exert a pivotal function in promoting angiogenesis to form fully developed blood vessels, while TIMP1 is seen as the tissue inhibitor of MMP-9 [194]. Additionally, endoglin, a TGF-β co-receptor, is instrumental in maintaining the equilibrium of signaling cascades between TGF-β/ALK1/Smad1/5 and ALK5/Smad2/3, which effectively inhibits cell motility and division in physiological conditions viaSmad2/3. In semaxanib-treated HUVECs, EGCG demonstrates a marked inhibitory activity on the upregulation of endoglin and decreases the levels of phosphorylated Smad1, thereby suppressing angiogenic capacity [195]. It also regulates the expression of multiple genes or proteins participating in angiogenesis, including TNFAIP2, EFNA1, PDGFA, CXCL6, IFN-β1, ID1, THBS-1, ANGPTL4, IL-1β, TGF-β2, and CCL2. These mediate proliferative, adhesion, invasion, and migratory actions of endothelial cells in human cervical cancer cells (HeLa) [196]. EGCG prevents cell migration toward VEGF in EPCs and TECs, but not in NEC. This corresponds to the downregulation of MMP-9 expression, inhibition of Akt phosphorylation in TEC, and suppression of VEGF-triggered migration of CD133/VEGFR2 cells into the bloodstream [197]. Moreover, EGCG blocks DNA replication, cellular proliferation, phosphorylation of ERK1/2, VEGFR-1 and -2, and EGR1 mRNA level triggered by VEGF in HUVECs [198].

EGCG has also been shown to directly suppress VEGF expression, inhibiting VEGF-induced tumor growth, proliferation, migration, and angiogenesis in breast carcinoma [199]. In A549 cells, EGCG suppresses pulmonary carcinoma angiogenesis stimulated by IGF-I by reducing the level of HIF-1 and VEGF [200]. EGCG inhibits tumor angiogenesis in HUVECs by inhibiting MT1-MMP, which degrades collagen type I and subsequent MMP-2 activation [201]. It has also been shown to inhibit MMP-2 and MMP-9 in SK-N-BE human neuroblastoma and HT1080 human fibrosarcoma cells [202]. Considering that CXCL12 attracts infiltration of tumor-associated macrophages (TAMs), which are a significant source of VEGFA and vital chemoattractant for macrophages. A modified form of EGCG called peracetate-protected EGCG has been developed as a precursor drug, which reduces VEGFA secreted by cancer cells and by TAM in endometrial carcinoma by inhibiting the PI3K/AKT/mTOR/HIF1 pathway and infiltration of VEGFA-expressing TAMs viaCXCL12 mediation in stromal cells [203].

#### 3.2.5. Flavanones

Research has demonstrated that the flavonoid naringenin, which is abundant in tomatoes and oranges, can inhibit angiogenesis. ERRα serves as a pivotal regulator in preserving energy balance and fostering generation of mitochondria by engaging with PPAR γ coactivator-1α and 1β. According to the literature, one of the mechanisms through which naringenin exerts its antiangiogenic effect is relevant to inducing cell cycle stasis at the G0-G1 phase and promoting apoptosis, and suppressing the release of cytokines MCP-1, IL-6, and ICAM-1involved in inflammation, and directly inhibiting the tyrosine phosphorylation function of KDR and blocking phosphorylated procedure of Akt, FAK, and paxillin induced by VEGF, and downregulating ERRα transactivation and expression, consequently leading to the inhibition of VEGF production [134].

In a laboratory experiment conducted with rat aortic rings, the flavanone 5,3′-dihydroxy-6,7,8,4′-tetramethoxyflavanone (HLBT-001) sourced from *Tillandsia recurvata* (L.) L. exhibited dose-dependent antiangiogenic potential by suppressing capillary sprout and tube development [135].

Hesperetin (3′,5,7-trihydroxy-4′-methoxyflavanone) derived from citrus fruits exerted an anti-angiogenic effect by inhibiting the creation of tubular structures, cell movement, and the proliferation of endothelial cells in VEGF-stimulated HUVECs [204]. This effect was found to be linked to the limitation of the VEGFR2-mediated signalings of AKT, p38 MAPK, PI3K, and ERK [136]. Additionally, Hesperetin demonstrated an inhibition of neovascularization factors like bFGF, EGF, and VEGF, along with a downregulation of mRNA COX-2 expression in rat colon carcinogenesis [137].

Monocytes exert a notable influence in angiogenesis and immune response regulation. Their adherence is influenced by the enhanced level of cell adhesion proteins like ICAM-1 and VCAM-1, as well as elevated secretion of chemokines [205]. Didymin (5,7-dihydroxy-7-rutinoside-4′-methoxy-flavone), a flavone glycoside found in oranges, lemons, and mandarins, has been shown to inhibit the VCAM-1 and ICAM-1 levels, effectively preventing high-glucose-induced monocyte adhesion to endothelial cells [206]. Moreover, Didymin reduces ROS production, NF-κB activation, and E-selectin, ICAM-1, and VCAM-1production in HUVECs induced by VEGF [138].

Farrerol (6,8-dimethyl-4′,5,7-trihydroxy-flavone), extracted from Rhododendron dauricum L., exhibits anti-vascular growth characteristics via the restraint of endothelial cell growth, migration, and generation of tubules. This effect is achieved by downregulating the Akt/mTOR, ERK, and JAK2/STAT3 signaling pathways in both HUVECs and HMECs-1 [139].

#### 3.2.6. Chalcones

A chalcone glycoside, hydroxysafflower yellow A (HSYA) (2′,3′,4′,4-tetrahydroxy-3′,5′-di-*O*-β-D-glucopyranosyl-chalcone), was discovered in Carthamus tinctorius L. and has been shown to inhibit tumor angiogenesis. In a study using transplanted human gastric adenocarcinoma BGC-823 cells, HSYAreduced MVD, integrated optical density (IOD) and downregulated the mRNA expression of VEGF and bFGF, which are crucial growth factors involved in cancer angiogenesis [140,141]. The inhibitory effects of HSYA on tumor angiogenesis were further demonstrated by decreasing the expression of MMP-9 and bFGF, as well as MMP-9 mRNA, regulating the degradation of the blood vessel basilar membrane, blood vessel migration, and tumor vascularization in BGC-823 cells [142]. CD105 has been assessed as a marker for neovascularization in hepatocellular carcinoma (HCC) [207]. HSYA displayed an inhibitory effect on angiogenesis factor secretion by suppressing the expression of CD105, VEGFA, bFGF, and VEGFR1, in addition to the phosphorylated ERK and c-raf. It also inhibited the phosphorylated process of IκB and prevented degradation within the cytoplasm of IκB-α, causing reduced NF-κB transcriptional activity by suppressing the signaling of MAPK, NF-κB, and ERK. HSYA downregulated the transcription of genes like cyclinD1, c-myc, and c-Fos, known to be phosphorylated by activated ERK and translocated into the nucleus. This was achieved by reducing the expression of nuclear p65, increasing cytoplasmic levels of p65, blocking IκB phosphorylation, and preventing cytoplasmic degradation of IκB-α [143,208,209]. Furthermore, activation of the transcription factor 2 (ATF-2), an AP1 transcription factor family member, is responsible for regulating the transcriptional activation of target genes. Several studies signify that ATF2 mediates VEGF-stimulated angiogenic processes while also exerting an inhibitory effect on angiogenesis by negatively regulating Notch-related genes such as DLL4, HEY1, and NRARP [210]. HSYA reduced the COX-2, MMP-9, and MMP-2 levels in HepG2 cell lysates by inhibiting p38 MAPK phosphorylation [144].

Tumor-infiltrating EPCs contributes to resistance against anti-VEGF therapy by indirectly secreting TGF-β, HIF-1α, and VEGF as angiogenesis-promoting growth factors [211]. The enzymes of the CYP 4 family are responsible for catalyzing the conversion of diverse fatty acids by hydroxylation, including ARA. Among these hydroxylation products, 20-HETE as a principal product results from the ARA hydroxylation by CYP4A and acts as a key facilitator in angiogenesis mediated by VEGF [212]. FLA-16 (2,3′,4,4′-tetrahydroxy-3,5′-diprenylchalcone) is a compound derived from Glycyrrhiza glabra [213]. It has been observed that CYP450 4A-derived 20-hydroxyeicosatetraenoic acid, in conjunction with the induction of pro-angiogenic growth factors by tumor-associated macrophages (TAMs), promotes angiogenesis during anti-VEGF treatment. Additionally, by specifically targeting TAMs and EPCs viathe PI3K/Akt signaling pathway, FLA-16 effectively normalizes the vasculature in glioma, resulting in a considerable drop in both VEGF and TGF-β expression [145].

SKLB-M8, namely (*E*)-3-(3-amino-4-methoxyphenyl)-1-(5-methoxy-2,2-dimethyl-2H-chromen-8-yl)prop-2-en-1-one hydrochloride, which is derived from Millettiapachycarpa Benth, has demonstrated inhibitory effects on the proliferative and migratory activities of HUVECs by suppressing ERK1/2 activation. Moreover, treatment with SKLB-M8 has been shown to effectively reduce angiogenesis within alginate beads implanted in mice and significantly decrease the uptake of fluorescein isothiocyanate (FITC)-dextran in mouse models bearing melanoma B16F10 tumors [146].

Licochalcone A (LicA) has been detected in Glycyrrhiza inflata and shown to possess inhibitory effects on endothelial cell movement and tube-like structure creation, along with blood vessel formation. It achieves this by downregulating the activation of VEGFR2, VEGF-stimulated phosphorylation of carbon store regulator C (cSrc) and inhibiting angiogenic growth factors of IL-8 and IL-6 [147]. The proteolytic cleavage of extracellular matrix proteins is a crucial process in cancer cell migration. Licochalcone E (LicE) demonstrates effective inhibition of VEGF-A secretion, a crucial factor in tumor angiogenesis, and suppresses VEGF-R2 activation. Additionally, it induces a reduction in tumor angiogenesis and brings about alterations in the local tumor tissue environment. This includes inhibiting the infiltration of leukocytes into tumor tissues, reducing HIF-1α expression, as well as downregulating the proinflammatory enzymes COX-2 and iNOS in tumor tissues [148].

Flavokawain A (FKA, 2′-hydroxy,4,4′,6′-trimethoxy chalcone) and flavokawain B (FKB, 2′-hydroxy,4′,6′-dimethoxy chalcone), two chalcones, can be extracted from the Piper methysticum or Alpinia pricei [214]. Angiogenin is a ribonuclease enzyme known for its crucial role in neovascularization, particularly within embryo, post-birth, and uterine lining tissues. Continual expression of F3 can contribute to the onset of thrombosis associated with cancer. FKB significantly decreases the expression of numerous angiogenic-associated factors, such as VEGF, F3, TSP-2, SDF-1, angiogenin, serpin F1, and pentraxin 3 [149]. FOXM1 acts as a transcriptional regulator of promoting cell cycle advancement, while GLUT1 serves as a carrier for glucose uptake viaglycolysis, particularly in tumors and often connected to cancer phenotypes. FKB effectively impedes the production of vascular-like structures by downregulating several key factors, including MMP9, VEGF, GLUT1, and FOXM1, with a specific emphasis on angiogenesis [150]. ICAM-1 is a glycoprotein known for its involvement in immune response and tumorigenesis. FKA demonstrates effective inhibition of VEGF, GLUT1, and ICAM-1 expression in the mammary carcinoma, resulting in a significant suppression of new vascular formation. In an ex vivo model employing rat aortic rings, increasing doses of FKA effectively impede vessel outgrowth from the fragmented aorta [151]. Additionally, FKA likely exerts an anti-angiogenic effect by downregulating the androgen receptor in bladder cancer to some extent, resulting in a more pronounced reduction in tumor growth in male transgenic mice compared to females [152].

Isolated from Alpinia katsumadai or Alpinia conchigera, cardamonin, namely 2′,4′-dihydroxy-6′-methoxychalcone, shows inhibitory activity on the protein levels of VEGF, HIF-1α, HIF-2α, and ribosomal S6 kinase 1 (S6K1) in CoCl2-induced hypoxic SKOV3 cells. These effects were partially attributed to mTOR inhibition [153]. Furthermore, cardamonin demonstrates anti-angiogenic properties by suppressing VEGF-induced angiogenesis and attenuating the VEGF-stimulated ERK and AKT phosphorylation in both HUVECs and mouse aortic ring assay [154]. MicroRNAs (miRNAs) represent short non-coding RNAs, regulating genetic transcription by interacting with mRNA targets, notably within the 3′ untranslated region. They impact various endothelial cell functions like apoptosis, differentiation, and angiogenesis. In a recent study, the blocking effect of VEGF-triggered angiogenesis by cardamonin is performed by diminishing the miR-21 expression, resulting in reduced cell growth, movement, and blood vessel formation stimulated by VEGF [155].

Isoliquiritigenin (4,2′,4′-trihydroxychalcone), derived from shallot, bean sprouts, or Glycyrrhiza glabra L. [215,216], exhibits inhibitory effects on the level and functionality of PMA-induced MMP-2 and MT1-MMP. Furthermore, it enhances the production of TIMP, upregulated by PMA, in a biphasic manner, while suppressing the MAPK-dependent signaling cascades involving p38 and JNK [156]. Isoliquiritigenin significantly inhibitsHUVEC proliferation stimulated by VEGF. This inhibition encompasses various anti-angiogenic processes, including the suppression of the capacity of HUVECs to form tubes, invade, and migrate, as well as prevention of VEGF-induced sprouting in aortic rings. Molecular mechanism studies revealed that isoliquiritigenin promotes HIF-1α proteasome degradation, resulting in a significant inhibition of VEGF expression in mammary carcinoma. Moreover, isoliquiritigenin directly interacts with VEGFR2 to inhibit its kinase function. Another report demonstrates that isoliquiritigenin effectively suppresses the proliferation of breast cancer cells and the formation of new blood vessels, accompanied by a suppressed VEGF/VEGFR2 pathway [157]. Isoliquiritigenin inhibits tumor angiogenesis in adenoid cystic carcinoma (ACC) cells through the obstruction of mTOR-dependent VEGF expression. This effect is achieved by activating JNK and inactivating ERK, resulting in a remarkable reduction inmicrovascular density in grafted tumors [158]. Moreover, isoliquiritigenin consistently exhibits anti-angiogenic activity by inhibiting the phosphorylation of ERK1/2, suppressing FGF-induced in vivo angiogenesis in mice, as well as cell proliferation, migration, and angiogenesis [159]. The maintenance of avascularity is delicately regulated by maintaining a delicate equilibrium between various anti-angiogenic factors like PEDF, and pro-angiogenic factors like FGF-2 and HGF, in which PEDF, a constituent of the serpin family, regulates cell multiplication and enhances neuronal survival. Isoliquiritigenin downregulates VEGF expression while upregulating PEDF level [160].

The overexpression of TM4SF5, a protein, is a crucial factor in encouraging the proliferation of tumor cells and is consistently found in hepatocarcinoma patients, contributing to hightened expression of TM4SF5 connected to VEGF level and angiogenesis [217]. During TM4SF5-induced multilayer growth, TM4SF5 activates FAK by increasing phosphorylation at Tyr577 (pY577FAK) and enhances the expression and stability of p27Kip1 in the cytosol. These processes lead to the inactivation of RhoA and contribute to the development of elongated morphology and epithelial–mesenchymal transition (EMT), which are responsible for promoting multilayer growth. A synthetic chalcone compound called TSAHC has been shown to effectively suppresses TM4SF5-mediated EMT, impedes multilayer growth, inhibits migration and invasion, and prevents the onset of tumors, showing the decrease inpY577FAK and p27Kip1 levels [161].

Xanthohumol(XN), namely 2′,4′,4-trihydroxy-6′-methoxy-3′-prenylchalcone, present in beer or Humulus lupulus, exhibit antiangiogenic effects. This is believed to occur viaareduction inNF-κB activity in HUVECs, leading to the inhibition of viability, invasion, and formation of capillary-like structures in endothelial cells [162,218]. XN demonstrates a dual anti-cancer effect by effectively suppressing NF-κB activity and IL1β expression in MCF7 cells and endothelial cells, impacting both tumor cells and angiogenesis [163]. Furthermore, XN displays a more potent anti-angiogenic effect compared to EGCG, which is linked to the upregulation of AMPK phosphorylation and activity mediated by CAMMKβ, as well as a reduction in eNOS phosphorylation partly induced by AKT signaling inhibition [164]. The angiogenesis process in leukemias relies heavily on the activation of Akt/NF-κB [219]. Therefore, XN suppresses angiogenesis by reducing VEGF secretion in leukemic cells, interfering with the cascades inducing NF-κB activation, and inhibiting Akt phosphorylation via suppression of Akt/NF-κB signaling in U937 chronic myelogenous leukemia [113], similar to the observed inhibitory effects in HUVECs [114]. Additionally, XN regulates the anti-angiogenic effects of NF-κB by suppressing the level of MMP-9, VEGF, and ICAM-1 stimulated by TNF in chronic myelogenous leukemia [115]. In the case of pancreatic cancer, XN blocks the induction of NF-κB to suppress angiogenesis, along with the subsequent expressions of VEGF and interleukin-8 (IL-8) [116].

Xanthoangelol, a natural chalcone also known as 2′,4,4′-trihydroxy-3′-[(*E*)-3,7-dimethyl-2,6-octadienyl]chalcone, was derived from Angelica keiskei [220]. It showed potent inhibitory effects on Lewis lung carcinoma-induced angiogenesis by blocking capillary-like tube formation and the interaction of HUVECs with VEGF [117].

Butein (3,4,2′,4′-tetrahydroxychalcone), a derivative from plants like Dalbergia odorifera, has revealed effective inhibition effects on cell proliferation, cell movement, and tubular structure induced by serum and VEGF in EPCs viathe downregulation of Akt and mTOR phosphorylation, as well as their major downstream effectors p70S6K, 4E-BP1, and eIF4E [118]. Additionally, butein exhibits anti-angiogenic properties by inhibiting NF-κB activity, as demonstrated in other studies. It specifically targets NF-κB, leading to the suppression of PMA- and TNF-α-stimulated MMP-9 and VEGF expression in prostatic carcinoma [119].

Tumor angiogenesis comprises a multifaceted process encompassing multiple factors and steps, including cellular expansion, migration, and degradation of the extracellular matrix. Many malignant solid tumors heavily rely on vascular supply and are typically associated with cascades of vascular dysplasia. The balance between angiogenesis promotion and inhibition factors plays a crucial role in this dependence [221].

**Figure 4:**
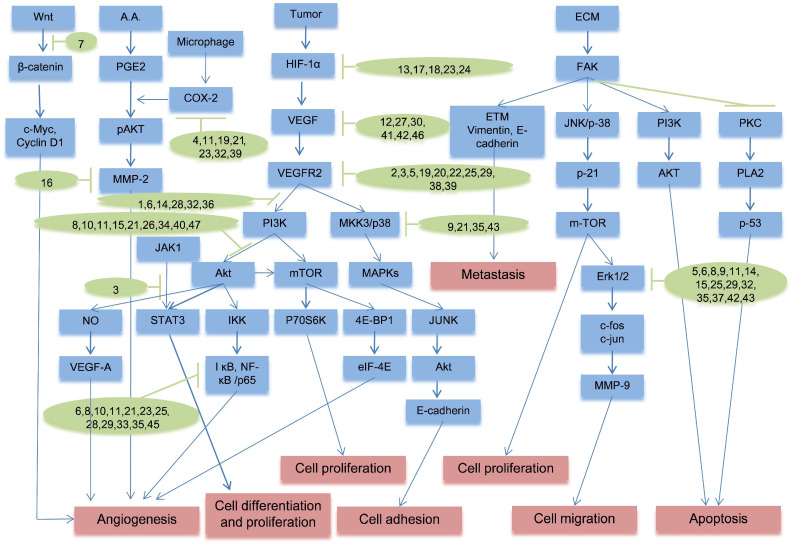
Partial mechanism of anti-angiongenic flavonoids in cancer (The blue represents the relative targets or pathways; the green represents the relative chemical compounds; the red represents the effects triggered by the targets or pathways).

As shown in Figure 4, there is a main pathway involving the proteins of HIF-1α, VEGF, VEGFR2, PI3K, and AKT, relating to the tumor anti-angiogenesis, including the most flavonoids in our findings. HIF-1α, an oxygen-dependent transcription factor, is highly expressed in tumor cells under hypoxic conditions, contributing significantly to tumorigenesis, development, invasion, metastasis, and apoptosis [222]. As shown in Figure 4, compounds **13**, **17**, **18**, **23**, and **24** exert anti-tumor angiogenic effects by inhibiting HIF-1. VEGF, the most powerful and specific factor for promoting tumor angiogenesis known to date, enhances endothelial cell proliferation, migration, and growth upon combination with VEGF [223]. VEGF is a crucial downstream target of HIF-1α, as its expression is upregulated by HIF-1α, ultimately leading to the formation of tumor angiogenesis, which can be inhibited by compounds **12**, **27**, **30**, **41**, **42** and **46** in Figure 4 [224]. Notably, HIF-1α also participates in cancer tissue growth, invasion, and metastasis, further emphasizing the importance of blocking the HIF-1α/VEGF pathway to effectively inhibit tumor angiogenesis [225].

VEGFR2 serves as a dominant mediator of both physiological and pathological angiogenesis [226]. HIF-1α enables VEGFR2 activation by binding to the hypoxia response element situated within the VEGF gene’s promoter region [227]. Therefore, compounds **2**, **3**, **5**, **19**, **20**, **22**, **25**, **29**, **38**, **39** in Figure 4 play a role of VEGFR2 inhibitor. The PH domain of AKT interacts with and binds to PI3K, leading to AKT’s structural alterations followed by phosphorylation. After activation, AKT relocates from the cytoplasm to the cellular membrane. This cascade facilitates the direct or indirect activation of downstream molecular proteins, including NF-κB and mTOR [228]. Akt is involved in controlling normal blood vessel formation and pathological angiogenesis, and its activation alone can stimulate VEGF expression in human cancer cells. mTOR, an essential protein kinase within the PI3K/Akt pathway, governs various critical cellular functions; its dysregulation has been implicated in tumorigenesis [229]. Once PI3K/Akt is activated, it can trigger mTOR activation, consequently elevating HIF-1α and VEGF expression as a pivotal player in the growth and formation of blood vessels by endothelial cells [230]. To some degree, compounds **1**, **6,14**, **28**, **32**, **36** and **8**, **10**, **11**, **15**, **21**, **26**, **34**, **40**, **47** in Figure 4 are related to the signal suppression PI3K/Akt pathway, whereas compounds **6**, **8**, **10**, **11**, **21**, **23**, **25**, **28**, **29**, **33**, **35**, and **45** entail the inhibition of NF-κB signal pathway.

In addition, many other important signal pathways are highly relevant to anti-angiogenic effects for future cancer therapy in Figure 4. COX-2, an inducible enzyme, is specifically generated in response to stimulation from associated cytokines, tumor inducers, and tumor genes [231]. The main pro-angiogenic activity of COX-2 occursviathe action of three ARA metabolites: PGI2, TXA2, and PGE2 [232]. Three eicosanoid products have a pivotal significance in downstream proangiogenic activities such as VEGF production, facilitation of blood vessel sprouting, migration, and tube development [233], improved endothelial cell survival via activating Akt production and increasing Bcl-2 level [234], stimulation of MMPs, initiation of EGFR-driven angiogenesis, and inhibition of interleukin-12 generation [235]. Compounds **4**, **11**, **19**, **21**, **23**, **32**, **39** in Figure 4 mitigate COX-2 in different ways and exerting the effect of inhibiting tumor neovascularization.

Wnt proteins are a family of signaling molecules that initiate a cascade of events within cells, influencing various developmental processes and maintaining tissue homeostasis. β-catenin transduces signals to the nucleus, activating Wnt-specific genes that regulate cell fate across various tissues [236]. The Wnt/β-catenin pathway activation is pivotal in driving angiogenesis by promoting growth of endothelial cells and facilitating neovascularization, interacting with various angiogenic proteins, like FGF and VEGF to influence their expression and activity [237]. Wnt signaling contributes to the establishment and maintenance of stable blood vessels and regulates the recruitment and function of pericytes, impacting vessel stability [238], which is achieved partly by compound **7** as an anti-angiogenic influence bysuppressing the Wnt/β-catenin pathway, as shown in in Figure 4.

JNK1 is a member of the MAPK family. In the context of angiogenesis, JNK1 signaling has been found to influence endothelial cell behaviors like cellular expansion, migration, and tubule creation for neovascularization [239]. STAT3 is a JNK1 downstream transcription factor controlling gene expression linked to cell survival and proliferation, and inflammation. In angiogenesis, STAT3 activation in endothelial cells regulates VEGF level, endothelial cell survival and migration, and blood vessel formation [240]. In some cancers, aberrant activation of JNK1/STAT3 pathways can promote tumor angiogenesis by initiating angiogenesis to facilitate the delivery of oxygen and nutrients to the tumor [241], which can be prevented by compound **3** as an anti-angiogenic agent in Figure 4.

The MAPK/AP-1 pathway holds significant importance in governing the expression of genes linked to metastasis. AP-1, a transcriptional regulator, is influenced by MAPKs including p38 MAPK, JNK1/2, and ERK1/2. MAPK phosphorylation regulation dictates the nuclear concentration of AP-1 subunits, which, in turn, determines the transcriptional activity of AP-1 [242]. ERK1/2 is pivotal in inducing immediate early genes (such as c-fos and c-jun) and orchestrates genetic transcriptional activation induced by growth factors, hormones, and cytokines [243]. The decline in genetic activity of COX-2, MMP-9, and MMP-3 corresponds to reduced phosphorylation levels of JNK1/2and ERK1/2, consequently diminishing nuclear c-Jun and c-Fos [242]. Furthermore, ERK1 and ERK2 collaborate in orchestrating endothelial cell proliferation and migration during angiogenesis [244]. However, compounds **5**, **6**, **8**, **9**, **11**, **14**, **15**, **26**, **29**, **32**, **35**, **37**, and **42** exhibit the capability to inhibit these processes in Figure 4.

## 4. Conclusions

The “tumor-starvation therapy” is a treatment method that cuts off the blood supply to the tumor, causing it to enter a state of starvation and thus limiting its growth. The main result of this research is to deeply explore the role and underlying molecular mechanisms of natural flavonoids in anti-angiogenesis. Additionally, this article systematically summarizes the structural characteristics and classification system of flavonoids, as well as the major signaling pathways involved in their function as plant-derived anti-angiogenic agents. These pathways include but are not limited to HIF-1α/VEGF/VEGFR2/PI3K/AKT, Wnt/β-catenin, JNK1/STAT3, and MAPK/AP-1, which may provide some new active ingredients or targets for future cancer treatment. At present, it is highly necessary to find non-toxic and cost-effective molecules based on or derived from the flavonoids’ structure for the advancement among cancer angiogenesis therapy, including luteonin, baicalein, apigenin, genistein, quercetin, silibinin, myricetin, kaempferol, fisetin, and EGCG, as deeply researched flavonoids in pharmacology. In addition, the validation of in vivo experiments also should receive high attention, not just cell experiments. It requires not only a significant amount of funding for investment but also enough experimental data through animal experiments and clinical trials, with multiple animals, models, and levels to determine the accuracy of targets and their treatment effectiveness.

## Supplementary Materials

The following supporting information can be downloaded at: https://www.mdpi.com/article/10.3390/molecules29071570/s1, Table S1. Antiangiogenic molecular mechanisms of different flavonoids.
