# Supplementary material for: Flavonoids with Anti-Angiogenesis Function in Cancer
Source: Molecules. 2024 Mar 31;29(7):1570. doi: 10.3390/molecules29071570 (PMC11013936; doi:10.3390/molecules29071570)
Supplement: Supplementary file 1 [file molecules-29-01570-s001.zip › molecules-2933459-supplementary.pdf]

Supplementary Material

Flavonoids with Anti-angiogenesis Function in Cancer

Table S1. Antiangiogenic molecular mechanisms of different flavonoids.

Type	Compounds (No.)	Cancer moder/cell line	Event	Mechanism	Referenc e
Flavone	Scutellarin(**1**)	HUVECs, CAM; colon cancer HCT116, LOVO, SW480 and HT29 cells	↓Tube formation and ephrinb2	Targeting ephrinb2 signaling	[1]
Flavone	Scutellarin(**1**)	Human oral cancer SAS cells	↓MMP-2, MMP-9, integrin $\alpha v\beta 6$	Possibly regulating transcription factor AP-1	[2]
Flavone	Scutellarin(**1**)	Melanoma A375 cells	↓p-Akt, p-mTOR, VEGF-A, MMP-2, MMP-9	Inhibiting PI3K/Akt/mTOR pathway	[3]
Flavone	Tricin(**2**)	HUVECs	↓VEGFR2 and VEGF	Reducing ROS; inhibiting HIF-1α accumulation	[4]
Flavone	Chrysin(**3**)	Prostate cancer DU145 cells	↓HIF-1α	Regulating PI3K/Akt signaling	[5]
Flavone	Chrysin(**3**)	HUVECs	↓gp130, sIL-6R, phosphorylated JAK1 and STAT3, VEGF	Downregulating JAK1/STAT3 pathway	[6]
Flavone	Chrysin(**3**)	HUVECs, CAM	↓VEGF, VEGFR2, L-6, IL-6R	Downregulating VEGF/VEGFR2 expression	[7]
Flavone	TTF1(**4**)	HepG-2-transplanted nude mice	↓VEGF, KDR, bFGF, COX-2, HIF-1α	Downregulating VEGF, KDR, bFGF, HIF-1α and COX-2	[8]
Flavone	Luteonin(**5**)	Breast cancer MCF-7 cells	↓AEG-1, MMP-2	Downregulating AEG-1 and MMP-2	[9]
Flavone	Luteonin(**5**)	BGC-823-transplanted nude mice	↓VEGF-A, MMP-9	Stimulating immune response and down-regulating VEGF-A and MMP-9	[10]
Flavone	Luteonin(**5**)	HUVECs, prostate cancer PC-3 cells	↓VEGFR2, Akt, ERK, mTOR, P70S6K, MMP-2 and MMP-9	Inhibiting AKT/ERK/mTOR/P70S6K/MMPs pathway	[11]
Flavone	Luteonin(**5**)	Human pancreatic carcinoma PANC-1, CoLo-357, BxPC-3 cells	↓VEGF secretion and expression; ↑phosphorylation level of JNK	Elevating JNK phosphorylation and inhibiting NF-κB-DNA binding activity	[12]
Flavone	Luteonin(**5**)	HMECs-1	↓PI3K, Akt, mTOR, p70S6k; ↓MMP-2, MMP-9, HSP90	Blocking Gas6/Axl pathway and PI3K/Akt/mTOR pathway	[13]
Flavone	Luteonin(**5**)	Mouse macrophage cells RAW264.7	↓VEGF, HIF-1α, MMP9	Downregulating HIF-1α and STAT3 signalling	[14]

Flavone	Luteonin(**5**)	HUVECs	↓MMP-2, IL-6Rα; ↑SOCS3	Modulating IL-6/STAT3 pathway	[15]
Flavone	Wogonin(**6**)	Breast cancer MCF-7 cells	↓HIF-1α, VEGF, MMP9; ↑PHD 1, 2, 3 and VHL;↓EGFR, Cdk4, survivin	Degrading HIF-1α protein	[16]
Flavone	Wogonin(**6**)	RPMI-8226 multiple myeloma cells	↓VEGF, PDGF, bFGF, VHL complex, VHL sumoylation and ubiquitination, c-Myc, HIF-1α	Modulating c-Myc/HIF-1α/VEGF signaling axis	[17]
Flavone	Wogonin(**6**)	Human gastric carcinoma in nude mice; HUVECs	↓ ERK1/2, AKT and p38	Inhibiting VEGFR2 phosphorylation	[18]
Flavone	Wogonin(**6**)	HUVECs	↓VEGF; PI3K and phosphorylation of Akt; ↑PTEN; ↓phosphorylation of IKK and IκB	Suppressing PI3K/Akt/NF-κB signaling	[19]
Flavone	Wogonoside(**7**)	Breast cancer MCF-7 cells	↓VEGF, Wnt3a; ↑GSK-3β, AXIN; promoting phosphorylation of β-catenin	Suppressing Wnt/β-catenin pathway	[20]
Flavone	Nobiletin(**8**)	HUVECs	↓ERK1/2, JNK, c-Jun, STAT3, proMMP-2, MMP-2, PA, Upa; ↑caspas3	Inhibiting VEGF- and bFGF-induced signaling and activating caspase pathway	[21]
Flavone	Nobiletin(**8**)	Human ovarian cancer OVCAR-3 and CP70 cells	↓VEGF, HIF-1α, AKT, NF-κB	Inhibiting Akt phosphorylation	[22]
Flavone	Nobiletin(**8**)	Breast cancer MCF-7 and T47D cells	↓VEGF, EGFR, bFGF, Src, FAK, STAT3, PXN; MMP2, MMP3, MMP9	Mediating Src/FAK/STAT3 signaling	[23]
Flavone	Oroxylin A(**9**)	HUVECs	↓phosphorylation of p38 MAPK, ERK1/2, Akt	Blocking KDR/Flk-1 phosphorylation	[24]
Flavone	Oroxyloside(**10**)	EA.hy926 human endothelial cell-like cell, NSCLC A549 cells	↓phosphorylated VEGFR2, Akt, p38, ERK, IKK, IκB, p65; ↑R-Ras, E-cadherin	Inhibiting Akt/MAPK/NF-κB pathway	[25]
Flavone	Baicalein(**11**)	NSCLC H-460, A549 and SKMES1 cells, xenografts	↓FGFR-2, VEGF, MMP1, TEK and ANGPT1	Partly mediated via VEGF and FGFR-2 signalling	[26]
Flavone	Baicalein(**11**)	HUVECs	↓MMP-2; inhibiting migration, proliferation	Reducing cell-associated MMP-2 activity	[27]

			and capillary formation of vascular endothelial cells		
Flavone	Baicalein(**11**)	Ovarian cancer OVCAR-3 and CP-70 cells	↓VEGF, c-Myc, HIF-α, NF-κB	Inhibiting VEGF, HIF-1α, cMyc and NFκB	[28]
Flavone	Baicalein(**11**)	BV2 murine microglial cells	↓HIF-1α, iNOS, COX-2, VEGF	Inhibiting ROS and PI 3K/Akt pathway	[29]
Flavone	Baicalein(**11**)	HUVECs	↓phosphorylation of VEGF2 and ERK, cyclin D, cyclin E, cdk-4, cdk-6, Rb; ↑p16, p21, p27 and p53	Regulating p53/Rb signaling; inhibiting ERK1/2 and p38 MAPK phospho- activation	[30]
Flavone	Baicalein(**11**)	HUVECs	↓AKT, ERK1/2, p38, TRAF6	Regulating TRAF6 mediated TLR4 pathway	[31]
Flavone	Genkwanin(**12**)	HUVECs	↓VEGF-induced invasion, tube formation	Inhibiting invasion and tube formation	[32]
Flavone	Acacetin(**13**)	Human ovarian cancer OVCAR-3 and A2780 cells	↓HIF-1α, VEGF and p-Akt	Inhibiting AKT/HIF-1α pathway	[33]
Flavone	Acacetin(**13**)	HUVECs	↓VEGF, eNOS, iNOS, MMP-2, bFGF	Suppressing the STAT-VEGF axis	[34]
Flavone	Apigenin(**14**)	HUVECs	↓E-cadherin, PECAM-1; ↑cleaved caspase-3, PARP, lamin A/C	Blocking the ERK signaling and ERK 1/2 survival signaling	[35]
Flavone	Apigenin(**14**)	Human ovarian cancer OVCAR-3 and A2780/CP70 cells	↓VEGF, HIF-1α, HDM2; ↑p53	Regulating PI3K/AKT/p70S6K1 and HDM2/p53 pathways	[36]
Flavone	Apigenin(**14**)	Human pancreatic cancer S2-013 and CD18 cells	↓HIF-1α, GLUT-1, VEGF	Downregulating HIF-1α, GLUT-1and VEGF	[37]
Flavone	Apigenin(**14**)	TRAMP mice	↓VEGF, uPA, MMP-2, MMP-9; ↑p-Akt and p-ERK1/2	Attenuating IGF-I/IGFBP-3 signaling	[38]
Flavone	Eupafolin(**15**)	Hepatocellular carcinoma cell lines HepG2 and Hep3B, HUVECs	↓Phosphorylation of ERK1/2, Akt and VEGF	Blocking VEGFR2 activation, and ERK1/2 and Akt phosphorylation	[39]
Flavone	HMM(**16**)	Standard reaction buffer	↓Cathepsins B and L	Inhibiting cathepsins B and L	[40]
Flavone	Eupatorin(**17**)	Human breast cancer MCF-7 and MDA-MB-231 cells	↓Bcl2L11, VEGFA, HIF1A genes; ↑Bax gene	Blocking the phospho-Akt pathway and cell cycle	[41]
Flavone	Sotetsuflavone	NSCLC A549 cells	↓VEGF, HIF-1α, MMP-9,	Modulating PI3K/AKT and	[42]

	(**18**)		MMP-13,TNF-α; ↑angiostatin	TNF-α/NF-κB pathways	
Flavone	Sotetsuflavone (**18**)	NSCLC A549 cells	↓TGF-β, STAT3, β-catenin; ↑endostatin, ZO-1	Inhibiting TGF-β, STAT3 and β-catenin; increasing endostatin and ZO-1	[43]
Flavone	Morusin(**19**)	Human HCC HepG2 and Hep3B cells, HUVECs	↓Bcl-2, VEGF, MMP2, MMP9, VEGFR2, IL-6 and phosphorylated STAT3; ↑Bax, active caspase-3	Attenuating IL-6/STAT3 signaling	[44]
Flavone	Morusin(**19**)	NSCLC A549 cells	↓Genes expression of VEGF and COX-2	Inhibiting VEGF and COX-2 genes	[45]
Isoflavone	Genistein(**20**)	Human prostate cancer PC-3 cells, HUVECs	↓VEGF, HIF-1α	Suppressing autocrine and paracrine signalings	[46]
Isoflavone	Genistein(**20**)	Human pancreatic carcinoma Capan-1, Capan-2, AsPc-1, PANC-1, and Mia PaCa-2 cells	↓VEGF, HIF-1	Inhibiting hypoxic activation of HIF-1 to downregulating VEGF gene expression	[47]
Isoflavone	Genistein(**20**)	HUVECs	↓MMP-1, MT1-MMP, pro-MMP-2, TIMP-1, TIMP-2, uPA, PAI-1	Preventing MMP-1 and uPA and the activation of pro-MMP-2, and modulating TIMP-1 and -2, and PAI-1	[48]
Isoflavone	Genistein(**20**)	Human bladder cancer RT4, T24, TSGH8301 cells	↓VEGF, PDGF-A, TF, uPA, MMP-2, MMP-9; ↑PAI-1, endostatin, angiostatin, TSP-1	Inhibiting VEGF, PDGF-A, TF, uPA, MMP-2 and MMP-9; upregulating PAI-1, endostatin, angiostatin and TSP-1	[49]
Isoflavone	Genistein(**20**)	Human prostate cancer PC3 cells	↓MMP-9, type IV collagenase, uPA, uPAR, protease M, PAR-2, VEGF, VEGFR, TGF-b, BPGF,LPA, TSP; ↑CTGF, CTAP	Downregulating type IV collagenase, uPA, uPAR,protease M, PAR-2, VEGF, VEGFR, TGF-b, BPGF,LPA, and TSP, and up-regulating CTGF and CTAP	[50]
Isoflavone	Genistein(**20**)	HUVECs	↓MMP-2, MMP-9, JNK, p38, PTK	Downregulating JNK and p38 activation	[51]
Flavonol	Quercetin(2**1**)	HUVECs, human prostate cancer PC3 cells	↓mTOR, Akt, p70S6K,	Regulating AKT/mTOR/P70S6K signaling	[52]
Flavonol	Quercetin(2**1**)	Human oral cancer SAS cells	↓MMP-2, MMP-9	Inhibiting NF-κB and MMP-2/MMP-9 signaling	[53]
Flavonol	Quercetin(2**1**)	Mammary carcinoma model	↓MVD, VEGF, Bfgf, H-ras protein	Inhibiting the H-ras protein synthesis, and VEGF and bFGF	[54]
Flavonol	Quercetin(2**1**)	HUVECs, human	↑TSP-1	Upregulating TSP-1	[55]

		prostate cancer PC3 cells			
Flavonol	Quercetin(**21**)	Lung cancer squamous carcinoma NCI-H157 cells	↓VEGF, p-Akt, p-STAT3	Suppressing STAT3 tyrosine phosphorylation, and Akt phosphorylation,	[56]
Flavonol	Quercetin(**21**)	Nasopharyngeal carcinoma NPC039 cells	↓VEGF, NF-κB	Suppressing NF-κB activity	[57]
Flavonol	Quercetin(**21**)	Bovine aortic endothelial cells	↓eNOS	Suppressing eNOS and early M-phase cell cycle arrest	[58]
Flavonol	Quercetin(**21**)	Human breast cancer MDA-MB-231 and MCF7 cells, HUVECs	↓COX-2 and PGE2 by inhibiting the binding of the transactivators CREB2, c-Jun, C/EBPβ, NF-κB	Inhibiting the p300 signaling and blocking the binding of multiple transactivators to COX-2 promoter	[59]
Flavonol	QODG(**22**)	HUVECs	↓VEGFR2, c-Src, FAK, ERK, AKT, mTOR, p70S6K	Suppressing VEGFR2-mediated signaling	[60]
Flavonol	Silibinin(**23**)	Human bladder transitional cell papilloma RT4 cells	↓Survivin; ↑p53	Downregulating survivin and increase p53	[61]
Flavonol	Silibinin(**23**)	Colon cancer LoVo cells	↓VEGF; ↑VEGFR1	Upregulating VEGFR-1	[62]
Flavonol	Silibinin(**23**)	Human prostate DU145 tumor xenografts	↓MVD, VEGF; ↑ IGFBP-3	Inhibiting VEGF and endothelial cell growth, and inducing apoptosis	[63]
Flavonol	Silibinin(**23**)	Female SKH-1 hairless mice	↓HIF-1α, STAT3, NF-κB, COX-2, iNOS, Tyr^{705} and Ser^{536}	Inhibiting NF-κB signaling	[64]
Flavonol	Silibinin(**23**)	Transgenic mouse model of prostate cancer	↓VEGF, VEGFR2, bFGF	Downregulating VEGF, VEGFR-2, and bFGF	[65]
Flavonol	Silibinin(**23**)	Human colorectal carcinoma HT29 xenograft tumor	↓NOS, NOS3, COX-1, COX-2, HIF-1α and VEGF	Downregulating NOS, COX, HIF-1α, and VEGF expression	[66]
Flavonol	Silibinin(**23**)	HUVECs	↓MMP-2, survivin; ↑Kip1/p27, Cip1/ p21, p53	Downregulating survivin; inhibiting Akt and NF-kB signaling, and MMP-2 secretion	[67]
Flavonol	Silibinin(**23**)	Human cervical (HeLa) and hepatoma (Hep3B) cells	↓HIF-1α, VEGF	Inhibiting PI3K/Akt signaling	[68]
Flavonol	Silibinin(**23**)	Human breast cancer MCF-7 cells	↓MMP-9, VEGF	Inactivating the Raf/MEK/ERK pathway	[69]

Flavonol	Myricetin(**24**)	SKH-1 hairless mouse skin tumorigenesis model	↓HIF-1α, MMP-9, MMP-13	Suppressing PI-3 kinase activity and attenuating Akt/p70S6K phosphorylation	[70]
Flavonol	Myricetin(**24**)	Ovarian cancer A2780/CP70 and OVCAR-3 cells	↓VEGF, p-Akt, p-70S6K, HIF-1α	Modulating Akt/p70S6K/HIF-1α/VEGF pathway and p21/HIF-1α/VEGF	[71]
Flavonol	Myricetin(**24**)	HUVECs	↓Cell migration, tube formation	Suppressing PI3K/Akt/mTOR signaling	[72]
Flavonol	Kaempferol(**25**)	Ovarian cancer OVCAR-3 and A2780/CP70 cells	↓VEGF, ERK phosphorelation, NF-κB, cMyc; ↑p21	Regulating ERK-NF-κB-cMyc-p21-VEGF pathway	[73]
Flavonol	Kaempferol(**25**)	Ovarian cancer OVCAR-3 and A2780/CP70 cells	↓VEGF, HIF-1α	Modulating Akt/HIF and ESRRA pathways	[74]
Flavonol	Kaempferol(**25**)	HUVECs	↓VEGFR2, p-Akt, p-p38	Inhibiting VEGFR2 expression, and VEGF and FGF pathways	[75]
Flavonol	Kaempferol(**25**)	HUVECs	↓VEGFR2, PI3K, AKT, mTOR, MEK1/2, ERK1/2	Targeting VEGFR2 and downregulating the PI3K/AKT, MEK and ERK pathways	[76]
Flavonol	Kaempferol(**25**)	HUVECs	↓HIF-1α, VEGFR2, ERK, p38, Akt, mTOR	Modulating ERK/p38 MAPK and PI3K/Akt/mTOR pathways	[77]
Flavonol	Rhamnazin(**26**)	HUVECs, human breast cancer MDA-MB-231 cells	↓VEGFR2, MAPK, AKT, STAT3; ↑PEDF	Regulating VEGF and PEDF; downregulating the VEGFR2/STAT3/MAPK/Akt pathway	[78,79]
Flavonol	Galangin(**27**)	HUVECs	↓VEGF	Downregulating CD44 and VEGF	[80]
Flavonol	Galangin(**27**)	Ovarian cancer A2780/CP70 and OVCAR-3 cells	↓VEGF, p-Akt, p-70S6K, HIF-1α	Modulating Akt/p70S6K/HIF-1α/VEGF pathway	[71]
Flavonol	Fisetin(**28**)	HUVECs, HT-1080 cells	↓MMP-14, MMP-1, MMP-3, MMP-7, MMP-9	Inhibiting the relational MMPs	[81]
Flavonol	Fisetin(**28**)	Colorectal cancer	↓MMP-8, MMP-13	Inhibiting MMP-8 and MMP-13	[82]
Flavonol	Fisetin(**28**)	HUVECs, prostate carcinoma DU145 and lung carcinoma A549 cells	↓Cyclin D1, survivin, VEGF, eNOS, iNOS, MMP-1, -3, -7, -9 and -14, Bcl-2; ↑caspases-3 and caspases-7, PARP, p53, p21, Bax	G1 phrase-G2/M arrest; Downregulating cyclin D1, survivin, VEGF, eNOS, iNOS, MMPs, Bcl-2; inducing p53 and p21, Bax expression and cleavage of caspases-3 and -7, and PARP	[83-84]
Flavonol	Fisetin(**28**)	Breast cancer 4T1 and JC cells	↓HO-1, MMP-2, MMP-9	Regulated by HO-1through transcription factor Nrf2	[85]
Flavonol	Fisetin(**28**)	Human prostate cancer PC-3 cells	↓MMP-2, MMP-9	Inactivating PI3K/Akt and JNK pathways and diminishing NF-κB and AP-1 DNA-binding activities	[86]

Flavonol	Fisetin(**28**)	Human cervical adenocarcinoma SiHa and CaSki cells	↓uPA, p-p38, NF-κB	Inhibiting p38 MAPK-dependent NF-κB pathway	[87]
Flavonol	Fisetin(**28**)	Pancreatic cancer cells	↓NF-κB, MMP9, EGFR, HER-2	Inhibiting DR3-mediated NF-κB activation	[88- 89]
Flavonol	Fisetin(**28**)	Prostate cancer	↓NF-κB, MAPK, Wnt, Akt, mTOR	Inhibiting NF-κB, MAPK, Wnt, Akt and mTOR	[90-91]
Flavanon ol	EGCG(**29**)	HUVECs	↓VEGFR-2, VEGF；	Inhibiting VEGF-induced VEGFR2 signaling	[92- 93]
Flavanon ol	EGCG(**29**)	Human endothelial cells	↓VEGF, VEGFR-1 and -2, ERK-1 and -2, MMP-2, MMP -9	Downregulating VEGF, VEGFR-1 and -2, ERK-1 and -2, MMP-2 and -9	[94-95]
Flavanon ol	EGCG(**29**)	SW620 colon cancer cells	↓MMP-9, NF-κB p-ERK1/2	Suppressing NF-κB and ERK1/2 signaling	[96]
Flavanon ol	EGCG(**29**)	NSCLC A549 cells	↓VEGF; ↑Endostatin	Increasing endostatin and suppressing VEGF	[97]
Flavanon ol	EGCG(**29**)	Colorectal cancer cells SW837	↓VEGF, HIF-1α, IGF-1, IGF-2, EGF, heregulin mRNAs, VEGFR2, p-VEGFR2, p-IGF-1 receptor, p-ERK, p-Akt	Suppressing HIF-1 and VEGF/VEGFR axis activation	[98]
Flavanon ol	EGCG(**29**)	NSCLC A549 and NCI-H460 cells	↓HIF-1α, VEGF, IL-8, CD31 and Akt	Inhibiting VEGF, IL-8, and CD31 and Akt activation	[99]
Flavanon ol	EGCG(**29**)	Human gastric cancer AGS cells	↓VEGF, p-STAT3	Inhibiting VEGF via suppressing STAT3 activity	[100]
Flavanon ol	EGCG(**29**)	Human gastric cancer SGC-7901 cells	↓VEGF, p-STAT3	Inhibing the VEGF secretion and STAT3activation	[101]
Flavanon ol	EGCG(**29**)	NSCLC A549 cells	↓ HIF-1α, VEGF, COX-2, p-Akt, p-ERK, vimentin protein, p53, β-catenin	Inhibiting angiogenesis and downregulating HIF-1α and VEGF	[102]
Flavanon ol	EGCG(**29**)	Human prostate carcinoma LNCaP cells	↓VEGF, uPA, angiopoietin 1 and 2, MMP-2 and -9; ↑TIMP1	Downregulating VEGF, uPA, angiopoietin 1 and 2, MMP-2 and -9 and elevating TIMP1	[103]
Flavanon ol	EGCG(**29**)	HUVECs	↓VEGF, endoglin, pSmad1	Downregulating endoglin/pSmad1 signaling	[104]
Flavanon ol	EGCG(**29**)	Human cervical tumour cells (HeLa)	↓TNFAIP2, EFNA1, PDGFA, CXCL6, THBS-1, TGF-β2, CCL2; ↑ANGPTL4, ID1, IFN-β1, IL-1β	Modulating the genes transcription involved in the angiogenic process	[105]
Flavanon ol	EGCG(**29**)	Endothelial cells, endothelial progenitor cells, bone marrow stromal cells	↓ MMP-9, p-Akt; suppressing the CD133+/VEGFR2+ cells into peripheral	Inhibiting Akt phosphorylation and VEGF-induced migration; downregulating MMP-9	[106]

			circulation		
Flavanonol	EGCG(**29**)	HUVECs	↓ VEGF-induced DNA synthesis, VEGFR-1 and -2, ERK1/2, EGR1	Inhibiting DNA synthesis and cell proliferation and the signal transduction pathways	[107]
Flavanonol	EGCG(**29**)	Mouse breast cancer E0771 cells	↓VEGF, HIF-1α, NF-κB	Inhibiting HIF-1α, NF-κB and VEGF	[108]
Flavanonol	EGCG(**29**)	NSCLC A549 cells	↓HIF-1α, VEGF	Downregulating HIF-1α and VEGF	[109]
Flavanonol	EGCG(**29**)	HUVECs	↓ MT1-MMP	Inhibiting MT1-MMP to block the endothelial cell invasion	[110]
Flavanonol	EGCG(**29**)	Human neuroblastoma SK-N-BE and human fibrosarcoma HT1080 cells	↓MMP-2, MMP-9	Inhibiting MMP-2 and MMP-9	[111]
Flavanonol	EGCG(**29**)	Human endometrial cancer cells AN3CA and RL95-2, stromal cells	↓VEGFA, HIF-1α, CXCL12	Inhibiting PI3K/AKT/mTOR/HIF1α pathway and downregulating CXCL12	[112]
Flavanones	Naringenin(**30**)	Human endothelial cells	↓ICAM-1, IL-6, MCP-1, VEGF, ERRα, KDR, p-Akt, p-paxillin	Mediating ERRα/VEGF/KDR signaling	[113]
Flavanones	HLBT-001(**31**)	Ex vivo rat aortic ring	↓ capillary sprout and tube formation	Not mentioned	[114]
Flavanones	Hesperetin(**32**)	HUVECs	↓p-PI3K, p-Akt, ERK, JNK, p38	Modulating PI3K/AKT, ERK and p38 MAPK signaling	[115]
Flavanones	Hesperetin(**32**)	Rat colon carcinogenesis	↓VEGF, bFGF, EGF and COX-2	Inhibiting angiogenic growth factors and COX-2 mRNA expression	[116]
Flavanones	Didymin(**33**)	HUVECs	↓ROS, NF-κB, and VCAM-1, ICAM-1, E-selectin	Preventing NF-κB and expression of adhesion molecules	[117]
Flavanones	Farrerol(**34**)	HUVECs, HMECs-1	↓p-ERK, p-Akt, p-mTOR, p-JAK2, p-STAT3, Bcl2, Bcl-xl	Downregulating Akt/mTOR, Erk and Jak2/Stat3 signals	[118]
Chalcone	HSYA(**35**)	Human gastric adenocarcinoma BGC-823 cells	↓ MVD, IOD	Inhibiting tumor vascularization	[119-120]
Chalcone	HSYA(**35**)	Human gastric adenocarcinoma BGC-823 cells	↓VEGF, MMP-9, bFGF, MMP-9 mRNA	Downregulating VEGF, bFGF and MMP-9	[121]
Chalcone	HSYA(**35**)	Murine hepatoma H22 cells	↓CD105, VEGFA, bFGF, VEGFR1, p-c-raf , p-ERK1/2, NF-κB, cyclinD1, c-myc, c-Fos	Blocking ERK/MAPK and NF-κB signaling	[122]

Chalcone	HSYA(**35**)	Murine hepatoma H22 cells and HepG2 human hepatocellular carcinoma cells	↓MMP-2, MMP-9, p-p38, ATF-2, COX-2	Inhibiting p38 MAPK phosphorylation	[123]
Chalcone	FLA-16(**36**)	C6 and U87 gliomas	↓20-HETE, VEGF, HIF-1α, TGF-β; ↑survival	Modulating PI3K/Akt signaling through inhibition of CYP4A	[124]
Chalcone	SKLB-M8(**37**)	HUVECs, B16F10 cells	↓p-ERK1/2	Decreasing ERK1/2 phosphorylation	[125]
Chalcone	LicA(**38**)	HUVECs	↓VEGFR2, IL-6, IL-8, cSrc	Blocking VEGF/VEGFR-2 signaling	[126]
Chalcone	LicE(**39**)	4T1 Mammary carcinoma cells	↓VEGFR2, VEGF-A, HIF-1α, COX-2, iNOS	Decreasing VEGFR2, VEGF-A, HIF-1α, COX-2, iNOS	[127]
Chalcone	FKB(**40**)	Two breast cancer cell MCF-7 and MDA-MB231	↓Angiogenin, F3, SDF-1, serpin F1, TSP-2	Reducing angiogenin, F3, SDF-1, serpin F1, and TSP-2	[128]
Chalcone	FKB(**40**)	MDA-MB231 cells, HUVECs	↓MMP9, VEGF, GLUT1, FOXM1	Suppressing the formation of vessels	[129]
Chalcone	FKA(4**1**)	MDA-MB231 cells	↓VEGF, GLUT1, ICAM1	Inhibiting new blood vessels	[130]
		Bladder tumor tissues in UPII-SV40T transgenic mouse	↓Androgen receptor	Downregulating the androgen receptor	[131]
Chalcone	Cardamonin(**42**)	SKOV3 cells	↓HIF-1α, HIF-2α, VEGF, S6K1	Inhibiting HIF-α and VEGF	[132]
Chalcone	Cardamonin(**42**)	HUVECs	↓p-ERK1/2, p-AKT	Regulating ERK1/2 and AKT signaling	[133]
Chalcone	Cardamonin(**42**)	HUVECs	↓miR-21	Downregulating miR-21	[134]
Chalcone	Isoliquiritigenin(**43**)	HUVECs	↓MMP-2, 1-MMP, JNK, p38 MAPK; ↑TIMP	Hampering MAPK signaling of JNK and p38	[135]
Chalcone	Isoliquiritigenin(**43**)	HUVECs, MCF-7 and MDA-MB-231cells	↓VEGF, HIF-1α, VEGFR-2, p-ERK1/2, p-Akt, p-JNK, eNOS, MMP-2	Inhibiting VEGF/VEGFR2 pathway	[136]
Chalcone	Isoliquiritigenin(**43**)	Adenoid cystic carcinoma cells ACC-M and ACC-2	↓MVD, VEGF, p-mTOR, p-S6, p-ERK1/2; ↑JNK, p-TSC-2	Dual activation of JNK and inhibition of ERK	[137]
Chalcone	Isoliquiritigenin(**43**)	HUVECs	↓p-ERK1/2, VEGF; ↑ PEDF	Inhibiting ERK1/2 and VEGF, and promoting PEDFexpression	[138-139]
Chalcone	TSAHC(**44**)	TM4SF5-null SNU449Cp or TM4SF5-expressing SNU449Tp stable cells	↓TM4SF5-mediated EMT, multilayer growth, migration,invasion and tumor formation ; p^{Y577}FAK, $p27^{Kip1}$	Disturbing protein-protein interaction between TM4SF5 and other membrane receptors	[140]
Chalcon	Xanthohumol(	HUVECs, SMC	↓NF-κB	Mitigating NF-κB activity	[141]

e	**45**)				
Chalcone	Xanthohumol(**45**)	MCF7 cells, endothelial cells	↓NF-κB, IL1β	Modulating NF-κB signalling	[142]
Chalcone	Xanthohumol(**45**)	HUVECs	↑p-AMPK CAMMKβ, LKB1; ↓eNOS, p-mTOR, p-70S6K, p-4EBP1	Inhibiting AMPK and AKT/mTOR pathway	[143]
Chalcone	Xanthohumol(**45**)	U937 cells, HUVECs	↓VEGF, MMP-2, MMP-9, p-p65, p-cytosolic IκBα, p-Akt	Inhibiting Akt/NF-kB signaling to decrease VEGF secretion	[144-145]
Chalcone	Xanthohumol(**45**)	KBM-5 cells	↓ICAM-1, MMP-9, VEGF	Inhibiting ICAM-1, MMP-9, and VEGF	[146]
Chalcone	Xanthohumol(**45**)	BxPC-3 and AsPC-1 cells	↓VEGF, IL-8, NF-κB	Suppressing NF-κB activity	[147]
Chalcone	Xanthoangelol (**46**)	HUVECs	↓VEGF	Inhibiting tube formation and the binding of VEGF to vascularendothelial cells	[148]
Chalcone	Butein(**47**)	EPCs	↓p-Akt, p-mTOR, p70S6K, 4E-BP1, eIF4E	Targeting the AKT/mTOR translation dependent signaling	[149]
Chalcone	Butein(**47**)	Human prostate cancer PC3 and DU145 cells	↓VEGF, MMP-9	Inhibiting NF-κB signaling	[150]

Note: "↓"indicates an increase in indicators; "↑" indicates an decrease in indicators.

References

1. Zhu, P. T.; Mao, M.; Liu, Z. G.; Tao L.; Yan B.C. Scutellarin suppresses human colorectal cancer metastasis and angiogenesis by targeting ephrinb2. *Am. J. Transl. Res.* **2017**, *9*, 5094. https://pubmed.ncbi.nlm.nih.gov/29218107/
2. Li, H.X.; Huang, D.Y.; Gao, Z.X.; Chen, Y.; Zhang, L.; Zheng, J.-H. Scutellarin inhibits the growth and invasion of human tongue squamous carcinoma through the inhibition of matrix metalloproteinase-2 and-9 and αvβ6 integrin. *Int. J. Oncol*. **2013**, *42*, 1674-1681. https://doi.org/10.3892/ijo.2013.1873
3. Li, C. Y.; Wang, Q.; Wang, X.M.; Li, G.X.; Wei, X.L. Scutellarin inhibits the invasive potential of malignant melanoma cells through the suppression epithelial-mesenchymal transition and angiogenesis via the PI3K/Akt/mTOR signaling pathway[J]. *Eur. J. Pharmacol*. **2019**, *858*, 172463. https://doi.org/10.1016/j.ejphar.2019.172463
4. Han, J. M.; Kwon, H. J.; Jung, H. J. Tricin, 4', 5, 7-trihydroxy-3', 5'-dimethoxyflavone, exhibits potent antiangiogenic activity in vitro. *Int. J. Oncol*. **2016**, *49*, 1497-1504. https://doi.org/10.3892/ijo.2016.3645
5. Fu, B.; Xue, J.; Li, Z.; Shi, X.; Jiang, B.-H.; Fang, J. Chrysin inhibits expression of hypoxia-inducible factor-1alpha through reducing hypoxia-inducible factor-1alpha stability and inhibiting its protein synthesis. *Mol. Cancer Ther.* **2007**, *6*, 220-226. https://doi.org/10.1158/1535-7163.mct-06-0526
6. Lin, C.M.; Shyu, K.G.; Wang, B.W.; Chang, H.; Chen, Y.H.; Chiu, J.H. Chrysin suppresses IL-6-induced angiogenesis via down-regulation of JAK1/STAT3 and VEGF: An in vitro and in ovo approach. *J Agric Food Chem*. **2010**, *58*, 7082–7087. https://doi.org/10.1021/jf100421w
7. Lin, C.M.; Chang, H.; Li, S.Y.; Wu, I.H.; Chiu, J.H. Chrysin inhibits lipopolysaccharide-induced angiogenesis via down-regulation of VEGF/VEGFR-2 (KDR) and IL-6/IL-6R pathways. *Planta Med*. **2006**, *72*, 708-714. https://doi.org/10.1055/s-2006-931602
8. Liu, C.; Li, X. W.; Cui, L. M.; Li, L.C.; Chen, L.Y.; Zhang, X.-W. Inhibition of tumor angiogenesis by TTF1 from extract of herbal medicine. *World J. Gastroentero*. **2011**, *17*, 4875. https://doi.org/10.3748/wjg.v17.i44.4875
9. Jiang, Y.; Xie, K.P.; Huo, H.N.; Wang, L.M.; Zou, W.; Xie, M.J. Inhibitory effect of luteolin on the angiogenesis of chick chorioallantoic membrane and invasion of breast cancer cells via downregulation of AEG-1 and MMP-2. *Acta Physiol. Sin*. **2013**, *65*, 513–518. https://pubmed.ncbi.nlm.nih.gov/24129732/
10. Lu, X.Y.; Li, Y.H.; Xiao, X.W.; Li, X.B. Inhibitory effects of luteolin on human gastric carcinoma xenografts in nude mice and its mechanism. *Nat. Med. J .China*. **2013**, *93*,142–146. https://pubmed.ncbi.nlm.nih.gov/23648354/

11. Pratheeshkumar, P.; Son, Y.O.; Budhraja, A.; Wang, X.; Ding, S.-Z; Wang, L.; Hitron, A.; Lee, J.C.; Kim, D.; Divya, S.P.; Chen, G.; Zhang, Z.; Luo, J.; Shi, X.L. Luteolin inhibits human prostate tumor growth by suppressing vascular endothelial growth factor receptor 2-mediated angiogenesis. *PLoS One*. **2012**, *7*, e52279. https://doi.org/10.1371/journal.pone.0052279
12. Cai, X.; Lu, W.; Ye, T.; Lu, M.; Wang, J.; Huo, J.; Qian, S.-H.; Wang, X.-N.; Cao, P. The molecular mechanism of luteolin-induced apoptosis is potentially related to inhibition of angiogenesis in human pancreatic carcinoma cells. *Oncol. Rep*. **2012**, *28*, 1353–1361. https://doi.org/10.3892/or.2012.1914
13. Li, X.; Chen, M.; Lei, X.; Huang, M.; Ye, W.; Zhang, R.; Zhang, D. Luteolin inhibits angiogenesis by blocking Gas6/Axl signaling pathway. *Int. J. Oncol*. **2017**, *51*, 677-685. https://doi.org/10.3892/ijo.2017.4041
14. Fang, B.; Chen, X.; Wu, M.; Kong, H.; Chu, G.; Zhou, Z.; Zhang, C.; Chen, B. Luteolin inhibits angiogenesis of the M2-like TAMs via the downregulation of hypoxia inducible factor-1α and the STAT3 signalling pathway under hypoxia. *Mol. Med. Rep*. **2018**, *18*, 2914-2922. https://doi.org/10.3892/mmr.2018.9250
15. Lamy, S.; Akla, N.; Ouanouki, A.; Lord-Dufour, S.; Beliveau, R. Diet-derived polyphenols inhibit angiogenesis by modulating the interleukin-6/STAT3 pathway. *Exp. Cell Res*. **2012**, *318*, 1586–1596. https://doi.org/10.1016/j.yexcr.2012.04.004
16. Song, X.M.; Yao, J.Y.; Wang, F.; Zhou, M.; Zhou, Y.X.; Wang, H.; Wei, L.B.; Zhao, L.; Li, Z.Y.; Lu, N.; Guo, Q. L. Wogonin inhibits tumor angiogenesis via degradation of HIF-1α protein. *Toxicol. Appl. Pharmacol*. **2013**, *271*, 144–155. https://doi.org/10.1016/j.taap.2013.04.031
17. Fu, R.; Chen, Y.; Wang, X.P.; An, T.; Tao, L.; Zhou, Y.X.; Huang, Y.-J.; Chen, B.-A.; Li, Z.Y.; You, Q.D.; Guo, Q.L.; Wu, Z.Q. Wogonin inhibits multiple myeloma-stimulated angiogenesis via the c-Myc/VHL/HIF-1α signaling axis. *Oncotarget*. **2016**, *7*, 5715–5727. https://doi.org/10.18632/oncotarget.6796
18. Lu, N.; Gao, Y.; Ling, Y.; Chen, Y.; Gu, H.Y.; Qi, Q.; Liu, W.; Wang, X.T.; You, Q.D.; Guo, Q.L. Wogonin suppresses tumor growth in vivo and VEGF-induced angiogenesis through inhibiting tyrosine phosphorylation of VEGFR2. *Life Sci*. **2008**, *82*, 956-963. https://doi.org/10.1016/j.lfs.2008.02.013
19. Zhou, M.; Song, X.; Huang, Y.; Wei, L.; Li, Z.; You, Q.; Guo, Q.; Lu, N. Wogonin inhibits H_2O_2-induced angiogenesis via suppressing the PI3K/Akt/NF-κB signaling pathway. *Vasc. Pharmacol*. **2014**, *60*, 110-119. https://doi.org/10.1016/j.vph.2014.01.010
20. Huang, Y.; Zhao, K.; Hu, Y.; Zhou, Y.; Luo, X.; Li, X.; Wei, L.; Li, Z.; You, Q.; Guo, Q.; Lu, N. Wogonoside inhibits angiogenesis in breast cancer via suppressing the Wnt/β-catenin pathway. *Mol. Carcinogen*. **2016**, *55*, 1598-1612. https://doi.org/10.1002/mc.22412
21. Kunimasa, K.; Ikekita, M.; Sato, M.; Ohta, T.; Yamori, Y.; Ikeda, M.; Kuranuki, S.; Oikawa, T. Nobiletin, a citrus polymethoxyflavonoid, suppresses multiple angiogenesis-related endothelial cell functions and angiogenesis in vivo. *Cancer Sci*. **2010**, *101*, 2462–2469. https://doi.org/10.1111/j.1349-7006.2010.01668.x
22. Chen, J.; Chen, A.Y.; Huang, H.; Ye, X.; Rollyson,W.D.; Perry, H.E.; Brown, K.C.; Rojanasakul, Y.; Rankin, G.O.; Dasgupta, P.; Chen, Y.C. The flavonoid nobiletin inhibits tumor growth and angiogenesis of ovarian cancers via the Akt pathway. *Int. J. Oncol*. **2015**, *46*, 2629–2638. https://doi.org/10.3892/ijo.2015.2946
23. Sp, N.; Kang, D.Y.; Joung, Y.H.; Park, J.H.; Kim, W.S.; Lee, H.K.; Song, K.D.; Park, Y.M.; Yang, Y.M. Nobiletin inhibits angiogenesis by regulating Src/FAK/STAT3-mediated signaling through PXN in ER(+) breast cancer cells. *Int. J. Mol. Sci*. **2017**, 18. https://doi.org/10.3390/ijms18050935
24. Gao, Y.; Lu, N.; Ling, Y.; Chen, Y.; Wang, L.; Zhao, Q.; Qi, Q.; Liu, W.; Zhang, H.; You, Q.; Guo, Q. Oroxylin A inhibits angiogenesis through blocking vascular endothelial growth factor-induced KDR/Flk-1 phosphorylation. J. Cancer Res. Clin. **2010**, *136*, 667-675. https://doi.org/10.1007/s00432-009-0705-2
25. Zhao, K.; Li, X.; Lin, B.; Yang, D.; Zhou, Y.; Li, Z.; Guo, Q.; Lu, N. Oroxyloside inhibits angiogenesis through suppressing internalization of VEGFR2/Flk-1 in endothelial cells. *J. Cell Physiol*. **2018**, *233*, 3454-3464. https://doi.org/10.1002/jcp.26198
26. Cathcart, M.C.; Useckaite, Z.; Drakeford, C.; Semik, V.; Lysaght, J.; Gately, K.; O'Byrne, K.J.; Pidgeon, G.P. Anti-cancer effects of baicalein in non-small cell lung cancer in vitro and in vivo. *BMC Cancer.* **2016**, *16*, 1-13. https://doi.org/10.1186/s12885-016-2740-0
27. Liu, J.J.; Huang, T.S.; Cheng, W.F.; Lu, F.J. Baicalein and baicalin are potent inhibitors of angiogenesis: Inhibition of endothelial cell proliferation, migration and differentiation. *Int. J. Cancer* .**2003**, *106*, 559–565. https://doi.org/10.1002/ijc.11267
28. Chen, J.; Li, Z.; Chen, A.Y.; Ye, X.; Luo, H.; Rankin, G.O.; Chen, Y.C. Inhibitory effect of baicalin and baicalein on ovarian cancer cells. Int. *J. Mol. Sci.* **2013**, *14*, 6012-6025. https://doi.org/10.3390/ijms14036012
29. Hwang, K.Y.; Oh, Y.T.; Yoon, H.; Lee, J.; Kim, H.; Choe, W.; Kang, I. Baicalein suppresses hypoxia-induced HIF-1alpha protein accumulation and activation through inhibition of reactive oxygen species and PI3-kinase/Akt pathway in BV2 murine microglial cells. *Neurosci. Lett*. **2008**, *444*, 264-269. https://doi.org/10.1016/j.neulet.2008.08.057
30. Ling, Y.; Chen, Y.; Chen, P.; Hui, H.; Song, X.; Lu, Z.; Li, C.; Lu, N.; Guo, Q. Baicalein potently suppresses angiogenesis induced by vascular endothelial growth factor through the p53/Rb signaling pathway leading to G1/S cell cycle arrest. *Exp. Biol. Med*. **2011**, *236*, 851-858. https://doi.org/10.1258/ebm.2011.010395
31. Ling, Y.; Wang, L.; Chen, Y.; Feng, F.; You, Q.; Lu, N.; Guo, Q. Baicalein inhibits angiogenesis induced by lipopolysaccharide through TRAF6 mediated toll-like receptor 4 pathway. *Biomed. Prev. Nutr*. **2011**, *1*, 172–179. https://doi.org/10.1016/j.bionut.2011.06.013

32. Koirala, N. Metabolic engineering of Eescherichia colibl21 (de3) for the production of methylated/glycosylated flavonoids and their biological activities (Doctoral dissertation). **2017**. Sun Moon University, Republic of Korea. http://archive.nnl.gov.np:8080/handle/123456789/245
33. Liu, L.Z.; Jing, Y.; Jiang, L.L.; Jiang, X.E.; Jiang, Y.; Rojanasakul, Y.; Jiang, B.H. Acacetin inhibits VEGF expression, tumor angiogenesis and growth through AKT/HIF-1α pathway. *Biochem. Bioph.. Res. Co.* **2011**, *413*, 299–305. https://doi.org/10.1016/j.bbrc.2011.08.091
34. Bhat, T.A.; Nambiar, D.; Tailor, D.; Pal, A.; Agarwal, R.; Singh, R.P. Acacetin inhibits in vitro and in vivo angiogenesis and downregulates STAT signaling and VEGF expression. *Cancer Prev. Res*. **2013**, *6*, 1128–1139. https://doi.org/10.1158/1940-6207.capr-13-0209
35. Kim, H.J.; Ahn, M.-R. Apigenin suppresses angiogenesis by inhibiting tube formation and inducing apoptosis. *Nat. Prod. Commun*. **2016**, *11*, 1433-1436. https://doi.org/10.1177/1934578x1601101005
36. Fang, J.; Xia, C.; Cao, Z.; Zheng, J.Z.; Reed, E.; Jiang, B.H. Apigenin inhibits VEGF and HIF-1 expression via PI3K/AKT/p70S6K1 and HDM2/p53 pathways. *FASEB J*. **2005**, *19*, 342–353. https://doi.org/10.1096/fj.04-2175com
37. Melstrom, L.G.; Salabat, M.R.; Ding, X.Z.; Strouch, M.J.; Grippo, P.J.; Mirzoeva, S.; Pelling, J.C.; Bentrem, D.J. Apigenin down-regulates the hypoxia response genes: HIF-1α, GLUT-1, and VEGF in human pancreatic cancer cells. *J. Surg. Res.* **2011**, *167*, 173-181. https://doi.org/10.1016/j.jss.2010.10.041
38. Shukla, S.; MacLennan, G.T.; Fu, P.; Gupta, S. Apigenin attenuates insulin-like growth Factor-I signaling in an autochthonous mouse prostate cancer model. *Pharm. Res*. **2012**, *29*, 1506-1517. https://doi.org/10.1007/s11095-011-0625-0
39. Jiang, H.; Wu, D.; Xu, D.; Yu, H.; Zhao, Z.; Ma, D.; Jin, J. Eupafolin exhibits potent anti-angiogenic and antitumor activity in hepatocellular carcinoma. *Int. J. Biol. Sci*. **2017**, *13*, 701-711. https://doi.org/10.7150/ijbs.17534
40. de Novais, L.M.; de Arueira, C.C.; Ferreira, L.F.; Ribeiro, T.A.; Sousa Jr, P.T.; Jacinto, M.J.; de Carvalho, M.G.; Judice, W.A.; Jesus, L.O.; de Souza, A.A.; Torquato, H.F. 4′-Hydroxy-6, 7-methylenedioxy-3-methoxyflavone: A novel flavonoid from *Dulacia egleri* with potential inhibitory activity against cathepsins B and L. *Fitoterapia*. **2019**, *132*, 26-29. https://doi.org/10.1016/j.fitote.2018.08.005
41. Razak, N.A.; Abu, N.; Ho, W.Y.; Zamberi, N.R.; Tan, S.W.; Alitheen, N.B.; Long, K.; Yeap, S.K. Cytotoxicity of eupatorin in MCF-7 and MDA-MB-231 human breast cancer cells via cell cycle arrest, anti-angiogenesis and induction of apoptosis. *Sci. Rep*. **2019**, *9*, 1514. https://doi.org/10.1038/s41598-018-37796-w
42. Wang, S.; Yan, Y.; Cheng, Z.; Hu, Y.; Liu, T. Sotetsuflavone suppresses invasion and metastasis in non-small-cell lung cancer A549 cells by reversing EMT via the TNF-α/NF-κB and PI3K/AKT signaling pathway. *Cell Death Discov*. **2018**, *4*, 26. https://doi.org/10.1038/s41420-018-0026-9
43. Cheng, Z.K.; Wang, S.H.; Hu, Y.L.; Yan, Y.; Liu, T.X. Effects of sotetsuflavone on expression of endostatin, TGF-β, STAT3, β-catenin and ZO-1 in non-small cell lung cancer A549 cells. *TMR Cancer*. **2018**, *1*, 58-65. https://doi.org/10.53388/tmrc201800030
44. Gao, L.; Wang, L.; Sun, Z.; Li, H.; Wang, Q.; Yi, C.; Wang, X. Morusin shows potent antitumor activity for human hepatocellular carcinoma in vitro and in vivo through apoptosis induction and angiogenesis inhibition. *Drug Des. Dev. Ther*. **2017**, 1789-1802. https://doi.org/10.2147/dddt.s138320
45. Yin, X.L.; Lv, Y.; Wang, S.; Zhang, Y.Q. Morusin suppresses A549 cell migration and induces cell apoptosis by downregulating the expression of COX 2 and VEGF genes. *Oncol. Rep.* **2018**, *40*, 504-510. https://doi.org/10.3892/or.2018.6431
46. Guo, Y.; Wang, S.; Hoot, D.R.; Clinton, S.K. Suppression of VEGF-mediated autocrine and paracrine interactions between prostate cancer cells and vascular endothelial cells by soy isoflavones. *J. Nutr. Biochem.* **2007**, *18*, 408-417. https://doi.org/10.1016/j.jnutbio.2006.08.006
47. Büchler, P.; Reber, H.A.; Büchler, M.W.; Friess, H.; Lavey, R.S.; Hines, O.J. Antiangiogenic activity of genistein in pancreatic carcinoma cells is mediated by the inhibition of hypoxia-inducible factor-1 and the down-regulation of VEGF gene expression. *Cancer*. **2004**, *100*, 201-210. https://doi.org/10.1002/cncr.11873
48. Kim, M.H. Flavonoids inhibit VEGF/bFGF-induced angiogenesis in vitro by inhibiting the matrix-degrading proteases. *J. Cell Biochem*. **2003**, *89*, 529-538. https://doi.org/10.1002/jcb.10543
49. Su, S.J.; Yeh, T.M.; Chuang, W.J.; Ho, C.L.; Chang, K.L.; Cheng, H.L.; Liu, H.S.; Cheng, H.L.; Hsu, P.Y.; Chow, N.H. The novel targets for anti-angiogenesis of genistein on human cancer cells. *Biochem. Pharmacol*. **2005**, *69*, 307-318. https://doi.org/10.1016/j.bcp.2004.09.025
50. Li, Y.; Sarkar, F.H. Down-regulation of invasion and angiogenesis-related genes identified by cDNA microarray analysis of PC3 prostate cancer cells treated with genistein. *Cancer Lett.* **2002**, *186*, 157–164. https://doi.org/10.1016/s0304-3835(02)00349-x
51. Yu, X.; Zhu, J.; Mi, M.; Chen, W.; Pan, Q.; Wei, M. Anti-angiogenic genistein inhibits VEGF induced endothelial cell activation by decreasing PTK activity and MAPK activation. *Med. Oncol.* **2012**, *29*, 349-357. https://doi.org/10.1007/s12032-010-9770-2
52. Pratheeshkumar, P.; Budhraja, A.; Son, Y.O.; Wang, X.; Zhang, Z.; Ding, S.-Z.; Wang, L.; Hitron, A.; Lee, J.C.; Xu, M.; Chen, G.; Luo, J.; Shi, X.L. Quercetin inhibits angiogenesis mediated human prostate tumor growth by targeting VEGFR 2 regulated AKT/mTOR/P70S6K signaling pathways. *PLoS One*. **2012**, *7*, e47516. https://doi.org/10.1371/journal.pone.0047516
53. Lai, W.W.; Hsu, S.C.; Chueh, F.S.; Chen, Y.Y.; Yang, J.S.; Lin, J.P.; Lien, J.C.; Tsai, C.H.; Chung, J.G. Quercetin inhibits migration and invasion of SAS human oral cancer cells through inhibition of NF-κB and matrix metalloproteinase-2/-9 signaling pathways. *Anticancer Res.* **2013**, *33*, 1941–1950. https://pubmed.ncbi.nlm.nih.gov/23645742/

54. Kong, L.; Wu, K.; Lin, H. Inhibitory effects of quercetin on angiogenesis of experimental mammary carcinoma. *Chin. J. Clin. Oncol.* **2005**, *2*, 631–636. https://doi.org/10.1007/bf02739722
55. Yang, F.; Jiang, X.; Song, L.; Wang, H.; Mei, Z.; Xu, Z.; Xing, N. Quercetin inhibits angiogenesis through thrombospondin-1 upregulation to antagonize human prostate cancer PC-3 cell growth in vitro and in vivo. *Oncol. Rep.* **2016**, *35*, 1602–1610. https://doi.org/10.3892/or.2015.4481
56. Ansó, E.; Zuazo, A.; Irigoyen, M.; Urdaci, M.C.; Rouzaut, A.; Martínez-Irujo, J.J. Flavonoids inhibit hypoxia-induced vascular endothelial growth factor expression by a HIF-1 independent mechanism. *Biochem. Pharmacol.* **2010**, *79*, 1600-1609. https://doi.org/10.1016/j.bcp.2010.02.004
57. Huang, D.Y.; Dai, Z.R.; Li, W.-M.; Wang, R.G.; Yang, S.M. Inhibition of EGF expression and NF-κB activity by treatment with quercetin leads to suppression of angiogenesis in nasopharyngeal carcinoma. *Saudi. J. Biol. Sci.* **2018**, *25*, 826–831. https://doi.org/10.1016/j.sjbs.2016.11.011
58. Jackson, S.J.; Venema, R.C. Quercetin inhibits eNOS, microtubule polymerization, and mitotic progression in bovine aortic endothelial cells. *J. Nutr.* **2006**, *136*, 1178–1184. https://doi.org/10.1093/jn/136.5.1178
59. Xiao, X.; Shi, D.; Liu, L.; Wang, J.; Xie, X.; Kang, T.; Deng, W. Quercetin suppresses cyclooxygenase-2 expression and angiogenesis through inactivation of P300 signaling. *PLoS One*. **2011**, *6*, e22934. https://doi.org/10.1371/journal.pone.0022934
60. Lin, C.; Wu, M.; Dong, J. Quercetin-4'-O-β-D-glucopyranoside (QODG) inhibits angiogenesis by suppressing VEGFR2-mediated signaling in zebrafish and endothelial cells. *PLoS One.* **2012**, *7*, e31708. https://doi.org/10.1371/journal.pone.0031708
61. Singh, R.P.; Tyagi, A.; Sharma, G.; Mohan, S.; Agarwal, R. Oral silibinin inhibits in vivo human bladder tumor xenograft growth involving down-regulation of survivin. *Clin. Cancer Res*. **2008**, *14*, 300–308. https://doi.org/10.1158/1078-0432.ccr-07-1565
62. Yang, S.-H.; Lin, J.K.; Huang, C.J.; Chen, W.S.; Li, S.Y.; Chiu, J.H. Silibinin inhibits angiogenesis via Flt-1, but not KDR, receptor up-regulation. *J. Surg. Res*. **2005**, *128*, 140–146. https://doi.org/10.1016/j.jss.2005.04.042
63. Singh, R.P.; Sharma, G.; Dhanalakshmi, S.; Agarwal, C.; Agarwal, R. Suppression of advanced human prostate tumor growth in athymic mice by silibinin feeding is associated with reduced cell proliferation, increased apoptosis, and inhibition of angiogenesis. *Cancer Epidem. Biomar*. **2003**, *12*, 933-939. https://pubmed.ncbi.nlm.nih.gov/14504208/
64. Gu, M.; Singh, R.P.; Dhanalakshmi, S.; Agarwal, C.; Agarwal, R. Silibinin inhibits inflammatory and angiogenic attributes in photocarcinogenesis in SKH-1 hairless mice. *Cancer Res*. **2007**, *67*, 3483–3491. https://doi.org/10.1158/0008-5472.can-06-3955
65. Singh, R.P.; Raina, K.; Sharma, G.; Agarwal, R. Silibinin inhibits established prostate tumor growth, progression, invasion, and metastasis and suppresses tumor angiogenesis and epithelial-mesenchymal transition in transgenic adenocarcinoma of the mouse prostate model mice. *Clin. Cancer Res*. **2008**, *14*, 7773-7780. https://doi.org/10.1158/1078-0432.ccr-08-1309
66. Singh, R.P.; Gu, M.; Agarwal, R. Silibinin inhibits colorectal cancer growth by inhibiting tumor cell proliferation and angiogenesis. *Cancer Res*. **2008**, *68*, 2043–2050. https://doi.org/10.1158/0008-5472.can-07-6247
67. Singh, R.P.; Dhanalakshmi, S.; Agarwal, C.; Agarwal, R. Silibinin strongly inhibits growth and survival of human endothelial cells via cell cycle arrest and downregulation of survivin, Akt and NF-κB: implications for angioprevention and antiangiogenic therapy. *Oncogene*. **2005**, *24*, 1188-1202. https://doi.org/10.1038/sj.onc.1208276
68. García-Maceira, P.; Mateo, J. Silibinin inhibits hypoxia-inducible factor-1alpha and mTOR/p70S6K/4E-BP1 signaling pathway in human cervical and hepatoma cancer cells: implications for anticancer therapy. *Oncogene*. **2009**, *28*, 313. https://doi.org/10.1038/onc.2008.398
69. Kim, S.; Choi, J.H.; Lim, H.I.; Lee, S.K.; Kim, W.W.; Kim, J.S.; Kim, J.H.; Choe, J.H.; Yang, J.H.; Nam, S.J.; Lee, J.E. Silibinin prevents TPA-induced MMP-9 expression and VEGF secretion by inactivation of the Raf/MEK/ERK pathway in MCF-7 human breast cancer cells. *Phytomedicine*. **2009**, *16*, 573-580. https://doi.org/10.1016/j.phymed.2008.11.006
70. Jung, S.K.; Lee, K.W.; Byun, S.; Lee, E.J.; Kim, J.E.; Bode, A.M.; Dong, Z.; Lee, H.J. Myricetin inhibits UVB-induced angiogenesis by regulating PI-3 kinase in vivo. *Carcinogenesis*. **2010**, *31*, 911-917. https://doi.org/10.1093/carcin/bgp221
71. Huang, H.; Chen, A.Y.; Rojanasakul, Y.; Ye, X.; Rankin, G.O.; Chen, Y.C. Dietary compounds galangin and myricetin suppress ovarian cancer cell angiogenesis. *J. Funct. Foods.* **2015**, *15*, 464-475. https://doi.org/10.1016/j.jff.2015.03.051
72. Kim, G.D. Myricetin inhibits angiogenesis by inducing apoptosis and suppressing PI3K/Akt/mTOR signaling in endothelial cells. *J. Cancer Prev*. **2017**, *22*, 219-227. https://doi.org/10.15430/jcp.2017.22.4.219
73. Luo, H.; Rankin, G.O.; Juliano, N.; Jiang, B.H.; Chen, Y.C. Kaempferol inhibits VEGF expression and in vitro angiogenesis through a novel ERK-NFkappaB-cMyc-p21 pathway. *Food Chem.* **2012**, *130*, 321–328. https://doi.org/10.1016/j.foodchem.2011.07.045
74. Luo, H.; Rankin, G.O.; Liu, L.; Daddysman, M.K.; Jiang, B.H.; Chen, Y.C. Kaempferol inhibits angiogenesis and VEGF expression through both HIF dependent and independent pathways in human ovarian cancer cells. *Nutr. Cancer.* **2009**, *61*, 554–563. https://doi.org/10.1080/01635580802666281
75. Liang, F.; Han, Y.; Gao, H.; Xin, S.; Chen, S.; Wang, N.; Qin, W.; Zhong, H.; Lin, S.; Yao, X.; Li, S. Kaempferol identified by zebrafish assay and fine fractionations strategy from dysosma versipellis inhibits angiogenesis through VEGF and FGF pathways. *Sci. Rep*. **2015**, *5*, 14468. https://doi.org/10.1038/srep14468
76. Chin, H.K.; Horng, C.T.; Liu, Y.S.; Lu, C.C.; Su, C.Y.; Chen, P.S.; Chiu, H.Y.; Tsai, F.J.; Shieh, P.C.; Yang, J.S. Kaempferol inhibits angiogenic ability by targeting VEGF receptor-2 and downregulating the PI3K/AKT, MEK and ERK pathways in VEGF-stimulated human umbilical vein endothelial cells. *Oncol. Rep.* **2018**, *39*, 2351-2357. https://doi.org/10.3892/or.2018.6312

77. Kim, G.D. Kaempferol Inhibits Angiogenesis by Suppressing HIF-1α and VEGFR2 Activation via ERK/p38 MAPK and PI3K/Akt/mTOR Signaling Pathways in Endothelial Cells. *Prev. Nutr. Food Sci.* **2017**, *22*, 320-326. https://doi.org/10.3746/pnf.2017.22.4.320
78. Yu, Y.; Zhou, X.Z.; Ye, L.; Yuan, Q.; Freeberg, S.; Shi, C.; Zhu, P.W.; Bao, J.; Jiang, N.; Shao, Y. Rhamnazin attenuates inflammation and inhibits alkali burn-induced corneal neovascularization in rats. *RSC Adv*. 2018, *8*, 26696-26706. https://doi.org/10.1039/c8ra03159b
79. Yu, Y.; Cai, W.; Pei, C.G.; Shao, Y. Rhamnazin, a novel inhibitor of VEGFR2 signaling with potent antiangiogenic activity and antitumor efficacy. *Biochem. Biophys. Res. Commun.* 2015, *458*, 913-919. https://doi.org/10.1016/j.bbrc.2015.02.059
80. Chen, D.; Li, D.; Xu, X.B.; Qiu, S.; Luo, S.; Qiu, E.; Rong, Z.; Zhang, J.; Zheng, D. Galangin inhibits epithelial-mesenchymal transition and angiogenesis by downregulating CD44 in glioma. *J. Cancer.* **2019**, *10*, 4499. https://doi.org/10.7150/jca.31487
81. Park, J.H.; Jang, Y.J.; Choi, Y.J.; Jang, J.W.; Kim, J.H.; Rho, Y.K.; Kim, I.J.; Kim, H.J.; Leem, M.J.; Lee, S.T. Fisetin inhibits matrix metalloproteinases and reduces tumor cell invasiveness and endothelial cell tube formation. *Nutr. Cancer*. **2013**, *65*, 1192-1199. https://doi.org/10.1080/01635581.2013.828090
82. Arowosegbe, M.A.; Ogunleye, A.J.; Eniafe, G.O.; Omotuyi, O.I.; Ehima, V.O.; Metibemu, D.S.; Ogungbe, B.; Kanmodi, R.I.; Ogunmola, O.J. Applications of in silico methodologies in exploring the inhibitory potentials of fisetin on MMP-8 and MMP-13 in colorectal cancer progression. *Int. J. Drug Dev. Res*. **2017**, *9*, 09-15. https://scholar.google.com/scholar?cluster=15226939542408094674&hl=en&as_sdt=2005&sciodt=0,5
83. Bhat, T.A.; Nambiar, D.; Pal, A.; Agarwal, R.; Singh, R.P. Fisetin inhibits various attributes of angiogenesis in vitro and in vivo—implications for angioprevention. *Carcinogenesis*. **2012**, *33*, 385-393. https://doi.org/10.1093/carcin/bgr282
84. Park, J.H.; Jang, Y.J.; Choi, Y.J.; Jang, J.W.; Kim, J.H.; Rho, Y.K.; Lee, S.T. Fisetin inhibits matrix metalloproteinases and reduces tumor cell invasiveness and endothelial cell tube formation. *Nutr. Cancer*. **2013**, *65*, 1192-1199. https://doi.org/10.1080/01635581.2013.828090
85. Tsai, C.F.; Chen, J.H.; Chang, C.N.; Lu, D.Y.; Chang, P.C.; Wang, S.L.; Yeh, W.L. Fisetin inhibits cell migration via inducing HO-1 and reducing MMPs expression in breast cancer cell lines. *Food Chem. Toxicol*. **2018**, *120*, 528–535. https://doi.org/10.1016/j.fct.2018.07.059
86. Chien, C.-S.; Shen, K.-H.; Huang, J.-S.; Ko, S.-C.; Shih, Y.-W. Antimetastatic potential of fisetin involves inactivation of the PI3K/Akt and JNK signaling pathways with downregulation of MMP-2/9 expressions in prostate cancer PC-3 cells. *Mol. Cell. Biochem*. **2009**, *333*, 169–180. https://doi.org/10.1007/s11010-009-0217-z
87. Chou, R.H.; Hsieh, S.C.; Yu, Y.L.; Huang, M.H.; Huang, Y.C.; Hsieh, Y.H. Fisetin inhibits migration and invasion of human cervical cancer cells by down-regulating urokinase plasminogen activator expression through suppressing the p38 MAPK-dependent NF-κB signaling pathway. *PLoS One.* **2013**, *8*, e71983. https://doi.org/10.1371/journal.pone.0071983
88. Murtaza, I.; Adhami, V.M.; Hafeez, B.B.; Saleem, M.; Mukhtar, H. Fisetin, a natural flavonoid, targets chemoresistant human pancreatic cancer AsPC-1 cells through DR3-mediated inhibition of NF-κB. *Int. J. Cancer*. **2009**,*125*, 2465-2473. https://doi.org/10.1002/ijc.24628
89. Basse`res D.S.; Baldwin A.S. Nuclear factor-kappaB and inhibitor of kappaB kinase pathways in oncogenic initiation and progression.*Oncogene*. **2006**, *25*, 6817-6830. https://doi.org/10.1038/sj.onc.1209942
90. N Syed, D.; M Adhami, V.; Imran Khan, M.; Mukhtar, H. Inhibition of Akt/mTOR signaling by the dietary flavonoid fisetin. *Anti-Cancer Agents Med. Chem.* **2013**, *13*, 995-1001. https://doi.org/10.2174/18715206113139990129
91. Aksamitiene, E.; Kiyatkin, A.; Kholodenko, B.N. Cross-talk between mitogenic Ras/MAPK and survival PI3K/Akt pathways: A fine balance. *Biochem. Soc. Trans*. **2012**, *40*, 139–146. https://doi.org/10.1042/bst20110609
92. Meng, F.; Abedini, A.; Plesner, A.; Verchere, C.B.; Raleigh, D.P. The flavanol (−)-epigallocatechin 3-gallate inhibits amyloid formation by islet amyloid polypeptide, disaggregates amyloid fibrils, and protects cultured cells against IAPP-induced toxicity. *Biochemistry*. **2010**, *49*, 8127-8133. https://doi.org/10.1021/bi100939a
93. Moyle, C.W.; Cerezo, A.B.; Winterbone, M.S.; Hollands, W.J.; Alexeev, Y.; Needs, P.W.; Kroon, P.A. Potent inhibition of VEGFR-2 activation by tight binding of green tea epigallocatechin gallate and apple procyanidins to VEGF: Relevance to angiogenesis. *Mol. Nutr. Food Res*. **2015**, *59*, 401–412. https://doi.org/10.1002/mnfr.201400478
94. Neuhaus, T.; Pabst, S.; Stier, S.; Weber, A.A.; Schrör, K.; Sachinidis, A.; Vetter, H.; Ko, Y.D. Inhibition of the vascular-endothelial growth factor-induced intracellular signaling and mitogenesis of human endothelial cells by epigallocatechin-3 gallate. *Eur. J. Pharmacol*. **2005**, *483*, 223-227. https://doi.org/10.1016/j.ejphar.2003.10.029
95. Fassina, G.; Vene, R.; Morini, M.; Minghelli, S.; Benelli, R.; Noonan, D.M.; Albini, A. Mechanisms of inhibition of tumor angiogenesis and vascular tumor growth by epigallocatechin-3-gallate. *Clin. Cancer Res*. **2004**, *10*, 4865-4873. https://doi.org/10.1158/1078-0432.ccr-03-0672
96. Zhou, F.; Zhou, H.; Wang, T.; Mu, Y.; Wu, B.; Guo, D.L.; Zhang, X.M.; Wu, Y.. Epigallocatechin-3-gallate inhibits proliferation and migration of human colon cancer SW620 cells in vitro. *Acta Pharmacol. Sin*. 2012,33, 120-126. https://doi.org/10.1038/aps.2011.139
97. Sakamoto, Y.; Terashita, N.; Muraguchi, T.; Fukusato, T.; Kubota, S. Effects of epigallocatechin-3-gallate (EGCG) on A549 lung cancer tumor growth and angiogenesis. *Biosci. Biotechnol. Biochem*. **2013**, *77*, 1799-1803. https://doi.org/10.1271/bbb.120882
98. Shimizu, M.; Shirakami, Y.; Sakai, H.; Yasuda, Y.; Kubota, M.; Adachi, S.; Tsurumi, H.; Hara, Y.; Moriwaki, H. (-)-Epigallocatechin gallate inhibits growth and activation of the VEGF/VEGFR axis in human colorectal cancer cells. *Chem. Biol. Interact*. **2010**, *185*, 247–252. https://doi.org/10.1016/j.cbi.2010.03.036

99. He, L.; Zhang, E.; Shi, J.; Li, X.; Zhou, K.; Zhang, Q.; Le, A.D.; Tang, X. (-)-Epigallocatechin-3-gallate inhibits human papillomavirus (HPV)-16 oncoprotein-induced angiogenesis in non-small cell lung cancer cells by targeting HIF-1α. Cancer *Chemother. Pharmacol.* **2013**, *71*, 713–725. https://doi.org/10.1007/s00280-012-2063-z
100. Zhu, B.H.; Chen,H.Y.; Zhan,W.H.;Wang,C.Y.; Cai, S.R.;Wang, Z.; Zhang,C.H.;He, Y.L. (-)-Epigallocatechin-3-gallate inhibits VEGF expression induced by IL-6 via Stat3 in gastric cancer. *World J. Gastroenterl*. **2011**, *17*, 2315–2325. https://doi.org/10.3748/wjg.v17.i18.2315
101. (154) Zhu, B.H.; Zhan, W.H.; Li, Z.R.; Wang, Z.; He, Y.L.; Peng, J.S.; Cai, S.R.; Ma, J.P.; Zhang, C.H. (-)-Epigallocatechin-3-gallate inhibits growth of gastric cancer by reducing VEGF production and angiogenesis. *World J. Gastroentero*. **2007**, *13*, 1162. https://doi.org/10.3748/wjg.v13.i8.1162
102. Shi, J.L.; Liu, F.; Zhang,W.Z.; Liu, X.; Lin, B.H.; Tang, X.D. Epigallocatechin-3-gallate inhibits nicotine-induced migration and invasion by the suppression of angiogenesis and epithelial-mesenchymal transition in non-small cell lung cancer cells. *Oncol. Rep.* **2015**, *33*, 2972–2980. https://doi.org/10.3892/or.2015.3889
103. Siddiqui, I.A.; Malik, A.; Adhami, V.M.; Asim, M.; Hafeez, B.B.; Sarfaraz, S.; Mukhtar, H. (2008). Green tea polyphenol EGCG sensitizes human prostate carcinoma LNCaP cells to TRAIL-mediated apoptosis and synergistically inhibits biomarkers associated with angiogenesis and metastasis. *Oncogene*. **2008**, *27*, 2055-2063. https://doi.org/10.1038/sj.onc.1210840
104. Chen, C.Y.; Lin, Y.J.; Wang, C.C.N.; Lan, Y.H.; Lan, S.J.; Sheu, M.J. Epigallocatechin-3-gallate inhibits tumor angiogenesis: involvement of endoglin/Smad1 signaling in human umbilical vein endothelium cells. *Biomed. Pharmacother*. **2019**, *120*, 109491. https://doi.org/10.1016/j.biopha.2019.109491
105. Tudoran, O.; Soritau, O.; Balacescu, O.; Balacescu, L.; Braicu, C.; Rus, M.; Gherman, C.; Virag, P.; Irimie, F.; Berindan-Neagoe, I. Early transcriptional pattern of angiogenesis induced by EGCG treatment in cervical tumour cells. *J. Cell. Mol. Med.* **2012**, *16*, 520-530. https://doi.org/10.1111/j.1582-4934.2011.01346.x
106. Ohga, N.; Hida, K.; Hida, Y.; Muraki, C.; Tsuchiya, K.; Matsuda, K.; Ohiro, Y.; Totsuka, Y.; Shindoh, M. Inhibitory effects of epigallocatechin-3 gallate, a polyphenol in green tea, on tumor-associated endothelial cells and endothelial progenitor cells. *Cancer Sci.* **2009**, *100*, 1963-1970. https://doi.org/10.1111/j.1349-7006.2009.01255.x
107. Neuhaus, T.; Pabst, S.; Stier, S.; Weber, A.A.; Schrör, K.; Sachinidis, A.; Vetter, H.; Ko, Y.D. Inhibition of the vascular-endothelial growth factor-induced intracellular signaling and mitogenesis of human endothelial cells by epigallocatechin-3 gallate. *Eur. J. Pharmacol*.**2004**, *483*, 223-227. https://doi.org/10.1016/j.ejphar.2003.10.029
108. Gu, J.W.; Makey, K.L.; Tucker, K.B.; Chinchar, E.; Mao, X.; Pei, I.; Thomas, E.Y.; Miele, L. EGCG, a major green tea catechin suppresses breast tumor angiogenesis and growth via inhibiting the activation of HIF-1α and NFκB, and VEGF expression. *Vasc. Cell*. **2013**, *5*, 1-10. https://doi.org/10.1186/2045-824x-5-9
109. Li, X.; Feng, Y.; Liu, J.; Feng, X.; Zhou, K.; Tang, X. (2013). Epigallocatechin-3-gallate inhibits IGF-I-stimulated lung cancer angiogenesis through downregulation of HIF-1α and VEGF expression. *J. Nutrigenet. Nutrige.* **2013**, *6*, 169-178. https://doi.org/10.1159/000354402
110. Yamakawa, S.; Asai, T.; Uchida, T.; Matsukawa, M.; Akizawa, T.; Oku, N. (−)-Epigallocatechin gallate inhibits membrane-type 1 matrix metalloproteinase, MT1-MMP, and tumor angiogenesis. *Cancer Lett*. **2004**, *210*, 47-55. https://doi.org/10.1016/j.canlet.2004.03.008
111. Garbisa, S.; Sartor, L.; Biggin, S.; Salvato, B.; Benelli, R.; Albini, A. Tumor gelatinases and invasion inhibited by the green tea flavanol epigallocatechin-3-gallate. *Cancer*. **2001**, *91*, 822-832. https://doi.org/10.1002/1097-0142(20010215)91:4<822::aid-cncr1070>3.0.co;2-g
112. Wang, J.; Man, G.C.W.; Chan, T.H.; Kwong, J.; Wang, C.C. A prodrug of green tea polyphenol (−)-epigallocatechin-3-gallate (Pro-EGCG) serves as a novel angiogenesis inhibitor in endometrial cancer. *Cancer Lett*. **2018**, *412*, 10-20. https://doi.org/10.1016/j.canlet.2017.09.054
113. Li, Q.; Wang, Y.; Zhang, L.; Chen, L.; Du, Y.; Ye, T.; Shi, X. Naringenin exerts anti-angiogenic effects in human endothelial cells: Involvement of ERRα/VEGF/KDR signaling pathway. *Fitoterapia*. **2016**, *111*, 78-86. https://doi.org/10.1016/j.fitote.2016.04.015
114. Lowe, H.I.; Toyang, N.J.; Watson, C.T.; Ayeah, K.N.; Bryant, J. HLBT-100: a highly potent anti-cancer flavanone from Tillandsia recurvata (L.) L. *Cancer Cell Int*. **2017**, *17*, 1-12. https://doi.org/10.1186/s12935-017-0404-z
115. Kim, G.D. Hesperetin inhibits vascular formation by suppressing of the PI3K/AKT, ERK, and p38 MAPK signaling pathways. *Prev. Nutr. Food Sci*. **2014**, *19*, 299–306. https://doi.org/10.3746/pnf.2014.19.4.299
116. Nalini, N.; Aranganathan, S.; Kabalimurthy, J. Chemopreventive efficacy of hesperetin (citrus flavonone) against 1,2-dimethylhydrazine-induced rat colon carcinogenesis. *Toxicol. Mech. Methods.*2012, *22*, 397-408. https://doi.org/10.3109/15376516.2012.673092
117. Shukla, K.; Sonowal, H.; Saxena, A.; Ramana, K.V. Didymin by suppressing NF-κB activation prevents VEGF-induced angiogenesis in vitro and in vivo. *Vascul. Pharmacol*. **2019**, *115*, 18-25. https://doi.org/10.1016/j.vph.2019.01.002
118. Dai, F.; Gao, L.; Zhao, Y.; Wang, C.; Xie, S. Farrerol inhibited angiogenesis through Akt/mTOR, Erk and Jak2/Stat3 signal pathway. *Phytomedicine*. **2016**, *23*, 686-693. https://doi.org/10.1016/j.phymed.2016.03.008
119. Xi, S.Y.; Zhang, Q.; Liu, C.Y.; Xie, H.; Yue, L.F.; Gao, X.M. (2012). Effects of hydroxy safflower yellow-A on tumor capillary angiogenesis in transplanted human gastric adenocarcinoma BGC-823 tumors in nude mice. *J. Tradit. Chin. Med.* **2012**, *32*, 243–248. https://doi.org/10.1016/s0254-6272(13)60019-9

120. Xi, S.Y.; Zhang, Q.; Xie, H.; Liu, L.; Liu, C.; Gao, X.; Zhang, J.; Wu, L.; Qian, L.; Deng, X. Effects of hydroxy safflor yellow A on blood vessel and mRNA expression with VEGF and bFGF of transplantation tumor with gastric adenocarcinoma cell line BGC-823 in nude mice. *China J. Chinese Mater. Med.* **2009**, *34*, 605–610. https://doi.CNKI:SUN:ZGZY.0.2009-05-028
121. Xi, S.; Zhang, Q.; Liu, C.; Xie, H.; Yue, L.; Zhao, Y.; Zang, B.; Gao, X. Effects of HSYA on expression of bFGF protein and MMP-9 in BGC-823 transplantation tumor of nude mice. *China J. Chinese Mater. Med*. **2010**, *35*, 2877–2881. https://doi.org/10.4268/cjcmm20102119
122. Yang, F.; Li, J.; Zhu, J.; Wang, D.; Chen, S.; Bai, X. Hydroxysafflor yellow A inhibits angiogenesis of hepatocellular carcinoma via blocking ERK/MAPK and NF-κB signaling pathway in H22 tumor-bearing mice. *Eur. J. Pharmacol.* **2015**, *754*, 105–114. https://doi.org/10.1016/j.ejphar.2015.02.015
123. Zhang, J.; Li, J.; Song, H.; Xiong, Y.; Liu, D.; Bai, X. Hydroxysafflor yellow A suppresses angiogenesis of hepatocellular carcinoma through inhibition of p38 MAPK phosphorylation. *Biomed. Pharmacother.* **2019**,*109*, 806-814. https://doi.org/10.1016/j.biopha.2018.09.086
124. Wang, C.; Li, Y.; Chen, H.; Zhang, J.; Zhang, J.; Qin, T.; Duan, C.; Chen, X.; Liu, Y.; Zhou, X.; Yang, J. Inhibition of CYP4A by a novel flavonoid FLA-16 prolongs survival and normalizes tumor vasculature in glioma. *Cancer Lett*. **2017**, *402*, 131-141. https://doi.org/10.1016/j.canlet.2017.05.030
125. Wang, J.; Yang, Z.; Wen, J.; Ma, F.; Wang, F.; Yu, K.; Tang, M.; Wu, W.; Dong, Y.; Cheng, X.; Nie, C. SKLB-M8 induces apoptosis through the AKT/mTOR signaling pathway in melanoma models and inhibits angiogenesis with a decrease of ERK1/2 phosphorylation. *J. Pharmacol. Sci*. **2014**, *126*, 198-207. https://doi.org/10.1254/jphs.14077fp
126. Kim, Y.H.; Shin, E.K.; Kim, D.H.; Lee, H.H.; Park, J.H.Y.; Kim, J.K. Antiangiogenic effect of licochalcone A. *Biochem. Pharmacol.***2010**, *80*, 1152-1159. https://doi.org/10.1016/j.bcp.2010.07.006
127. Kwon, S.J.; Park, S.Y.; Kwon, G.T.; Lee, K.W.; Kang, Y.H.; Choi, M.S.; Yun, J.W.; Jeon, J.H.; Jun, J.G.; Park, J.H.Licochalcone E present in licorice suppresses lung metastasis in the 4T1 mammary orthotopic cancer model.*Cancer Prev. Res*. **2013**, *6*, 603–613. https://doi.org/10.1158/1940-6207.capr-13-0012
128. Abu, N.; Mohamed, N.E.; Yeap, S.K.; Lim, K.L.; Akhtar, M.N.; Zulfadli, A.J.; Kee, B.B.; Abdullah, M.P.; Omar, A.R.; Alitheen, N.B. In vivo antitumor and antimetastatic effects of flavokawain B in 4T1 breast cancer cell-challenged mice. *Drug Des. Devel. Ther.* **2015**, *9*, 1401-1417. https://doi.org/10.2147/dddt.s67976
129. Abu, N.; Akhtar, M.N.; Yeap, S.K.; Lim, K.L.; Ho, W.Y.; Abdullah, M.P.; Ho, C.L.; Omar, A.R.; Ismail, J.; Alitheen, N.B. Flavokawain B induced cytotoxicity in two breast cancer cell lines, MCF-7 and MDA-MB231 and inhibited the metastatic potential of MDA-MB231 via the regulation of several tyrosine kinases in vitro. *BMC Complement. Altern. Med*. **2016**, *16*, 1-14. https://doi.org/10.1186/s12906-016-1046-8
130. Abu, N.; Akhtar, M.N.; Yeap, S.K.; Lim, K.L.; Ho, W.Y.; Zulfadli, A.J.; Omar, A.R.; Sulaiman, M.R.; Abdullah, M.P.; Alitheen, N.B. Flavokawain A induces apoptosis in MCF-7 and MDA-MB231 and inhibits the metastatic process in vitro. *PLoS One*. **2014**, *9*, e105244. https://doi.org/10.1371/journal.pone.0105244
131. Liu, Z.; Xu, X.; Li, X.; Liu, S.; Simoneau, A.R.; He, F.; Wu, X.R.; Zi, X. Kava chalcone, flavokawain A, inhibits urothelial tumorigenesis in the UPII-SV40T transgenic mouse model. *Cancer Prev. Res*. **2013**, *6*, 1365-1375. https://doi.org/10.1158/1940-6207.22524197.v1
132. Xue, Z.G.; Niu, P.G.; Shi, D.H.; Liu, Y.; Deng, J.; Chen, Y.Y. Cardamonin inhibits angiogenesis by mTOR downregulation in SKOV3 cells. *Planta Med*.**2016**, *82*, 70-75. https://doi.org/10.1055/s-0035-1557901
133. Tian, S.S.; Jiang, F.S.; Zhang, K.; Zhu, X.X.; Jin, B.; Lu, J.J.; Ding, Z.S. Flavonoids from the leaves of *Carya cathayensis* Sarg. inhibit vascular endothelial growth factor-induced angiogenesis. *Fitoterapia*. **2014**, *92*, 34–40. https://doi.org/10.1016/j.fitote.2013.09.016
134. Jiang, F.S.; Tian, S.S.; Lu, J.J.; Ding, X.H.; Qian, C.D.; Ding, B.; Ding, Z.S.; Jin, B. Cardamonin regulates miR-21 expression and suppresses angiogenesis induced by vascular endothelial growth factor. *BioMed. Res. Int.* **2015**,1–8. https://doi.org/10.1155/2015/501581
135. Kang, S.W.; Choi, J.S.; Choi, Y.J.; Bae, J.Y.; Li, J.; Kim, D.S.; Kim, J.L.; Shin, S.Y.; Lee, Y.J.; Kwun, I.S.; Kang, Y.H. Licorice isoliquiritigenin dampens angiogenic activity via inhibition of MAPK-responsive signaling pathways leading to induction of matrix metalloproteinases. *J. Nutr. Biochem*. **2010**, *21*,55–65. https://doi.org/10.1016/j.jnutbio.2008.10.004
136. Wang, Z.; Wang, N.; Han, S.; Wang, D.; Mo, S.; Yu, L. Dietary compound isoliquiritigenin inhibits breast cancer neoangiogenesis via VEGF/VEGFR-2 signaling pathway. *PLoS One*. **2013**, *8*, e68566. https://doi.org/10.1371/journal.pone.0068566
137. Sun, Z.J.; Chen, G.; Zhang, W.; Hu, X.; Huang, C.F.; Wang, Y.F.; Jia, J.; Zhao, Y.F. Mammalian target of rapamycin pathway promotes tumor-induced angiogenesis in adenoid cystic carcinoma: its suppression by isoliquiritigenin through dual activation of c-Jun NH2-terminal kinase and inhibition of extracellular signal-regulated kinase. *J. Pharmacol. Exp. Ther.* **2010**, *334*, 500-512. https://doi.org/10.1124/jpet.110.167692
138. He, W.; Li, Y. Phytochemical isoliquiritigenin inhibits angiogenesis ex vivo and corneal neovascularization in mice. *Altern. Integr. Med*. **2014**, *3*, 176. https://doi.org/10.4172/2327-5162.1000176
139. Jhanji, V.; Liu, H.; Law, K.; Lee, V.Y.-W.; Huang, S.-F.; Pang, C.-P.; Yam, G.H.-F. Isoliquiritigenin from licorice root suppressed neovascularization in experimental ocular angiogenesis models. *Brit. J. Ophthalmol*. **2011**, *95*, 1309–1315. https://doi.org/10.1136/bjophthalmol-2011-300110

140. Lee, S.A.; Lee, M.; Ryu, H.; Kwak, T.; Kim, H.; Kang, M.; Jung, O.; Kim, H.; Park, K.; Lee, J. Differential inhibition of transmembrane 4 L six family member 5 (TM4SF5)-mediated tumorigenesis by TSAHC and sorafenib. *Cancer Biol. Ther.* **2011**, *11*, 330–336. https://doi.org/10.4161/cbt.11.3.14099
141. Negrão, R.; Incio, J.; Lopes, R.; Azevedo, I.; Soares, R. Evidence for the effects of xanthohumol in disrupting angiogenic, but not stable vessels. *Int. J. Biomed. Sci*.**2007**, 3, 279. https://doi.org/10.59566/ijbs.2007.3279
142. Monteiro, R.; Calhau, C.; Silva, A.O.E.; Pinheiro-Silva, S.; Guerreiro, S.; Gärtner, F.; Azevedo, I.; Soares, R. Xanthohumol inhibits inflammatory factor production and angiogenesis in breast cancer xenografts. *J. Cell. Biochem*. **2008**, *104*, 1699-1707. https://doi.org/10.1002/jcb.21738
143. Gallo, C.; Dallaglio, K.; Bassani, B.; Rossi, T.; Rossello, A.; Noonan, D.M.; D'Uva, G.; Bruno, A.; Albini, A. Hop derived flavonoid xanthohumol inhibits endothelial cell functions via AMPK activation. *Oncotarget*.**2016**, *7*, 59917. https://doi.org/10.18632/oncotarget.10990
144. Dell'Eva, R.; Ambrosini, C.; Vannini, N.; Piaggio, G.; Albini, A.; Ferrari, N. AKT/NF-κB inhibitor xanthohumol targets cell growth and angiogenesis in hematologic malignancies. *Cancer*. **2007**,*110*, 2007-2011. https://doi.org/10.1002/cncr.23017
145. Albini, A.; Dell'Eva, R.; Vené, R.; Ferrari, N.; Buhler, D.R.; Noonan, D.M.; Fassina, G. Mechanisms of the antiangiogenic activity by the hop flavonoid xanthohumol: NF-κB and Akt as targets. *FASEB J*.**2006**, *20*, 527-529. https://doi.org/10.1096/fj.05-5128fje
146. Harikumar, K.B.; Kunnumakkara, A.B.; Ahn, K.S.; Anand, P.; Krishnan, S.; Guha, S.; Aggarwal, B.B. Modification of the cysteine residues in IκBα kinase and NF-κB (p65) by xanthohumol leads to suppression of NF-κB–regulated gene products and potentiation of apoptosis in leukemia cells. Blood, *J. Am. Soc. Hematol*. **2009**, *113*, 2003-2013. https://doi.org/10.1182/blood-2008-04-151944
147. Saito, K.; Matsuo, Y.; Imafuji, H.; Okubo, T.; Maeda, Y.; Sato, T.; Shamoto, T.; Tsuboi, K.; Morimoto, M.; Takahashi, H.; Ishiguro, H. Xanthohumol inhibits angiogenesis by suppressing nuclear factor-κB activation in pancreatic cancer. *Cancer Sci.* **2018**, *109*, 132-140. https://doi.org/10.1111/cas.13441
148. Kimura, Y.; Baba, K. Antitumor and antimetastatic activities of Angelica keiskei roots, part 1: Isolation of an active substance, xanthoangelol. *Int. J. Cancer.* **2003**, *106*, 429–437. https://doi.org/10.1002/ijc.11256
149. Chung, C.H.; Chang, C.H.; Chen, S.S.; Wang, H.H.; Yen, J.Y.; Hsiao, C.J.; Wu, N.L.; Chen, Y.L.; Huang, T.F.; Wang, P.C. Butein inhibits angiogenesis of human endothelial progenitor cells via the translation dependent signaling pathway. *Evid. Based Complement. Alternat. Med*. **2013**, 943187. https://doi.org/10.1155/2013/943187
150. Moon, D.O.; Choi, Y.H.; Moon, S.K.; Kim, W.J.; Kim, G.Y. Butein suppresses the expression of nuclear factor-kappa B-mediated matrix metalloproteinase-9 and vascular endothelial growth factor in prostate cancer cells. *Toxicol. Vitro.* **2010**, *24*, 1927-1934. https://doi.org/10.1016/j.tiv.2010.08.002
